# Overexpression of the grapevine PGIP1 in tobacco is associated with leaf cell wall pectin modification and glycoprotein changes following *Botrytis cinerea* infection

**DOI:** 10.3389/fpls.2026.1935736

**Published:** 2026-10-21

**Authors:** Florent Weiller, Jonatan U. Fangel, William G.T. Willats, Azeddine Driouich, Melané A. Vivier, John P. Moore

**Affiliations:** 1 Department of Viticulture and Oenology, South African Grape and Wine Research Institute, Stellenbosch University, Stellenbosch, South Africa; 2 Department of Plant and Environmental Sciences, University of Copenhagen, Copenhagen, Denmark; 3 School of Agriculture, Food and Rural Development, Newcastle University, Newcastle-upon-Tyne, United Kingdom; 4 Normandie Université, Laboratoire Glycobiologie et Matrice Extracellulaire Végétale EA 4358. Fédération de Recherche -Normandie Végétal- FED 4277, Université de Rouen Normandie, Rouen, France

**Keywords:** arabinogalactan protein, *Botrytis cinerea*, cell wall, extensin, *Nicotiana tabacum*, pectin, PGIP1, *Vitis vinifera*

## Abstract

**Introduction:**

Grapevine PGIP1 (polygalacturonase inhibiting protein 1) expressed in tobacco produces plants with enhanced resistance against *B. cinerea* infection. Grapevine PGIP1 has been shown to remodel leaf cell walls in transgenic tobacco in the absence of fungal infection. The associated dynamic cell wall changes linked to the mechanism of resistance have not been investigated in the context of fungal infection.

**Methods:**

Scanning electron microscopy was used to evaluate infection progression. Monosaccharide composition analysis was used to evaluate cell wall changes as infection progressed. Comprehensive microarray polymer profiling tracked polysaccharide and glycoprotein changes.

**Results:**

No difference was observed for lesions between wild-type and transgenic PGIP1 during the first 72 hpi. Rhamnogalacturonan I (RG-I) levels increased at the infection site. The cell wall glycoproteins, arabinogalactan proteins and extensin proteins, show higher signals in the lesion and adjacent tissue in response to *Botrytis cinerea* infection.

**Discussion:**

RG-I and cell wall protein reorganisation in grapevine PGIP1-expressing tobacco lines upon infection with *Botrytis cinerea* suggest cell wall remodelling enhances fungal resistance.

## 1 Introduction

The cell wall is an essential structure in plant growth and development as it provides mechanical strength for support and forms an effective physico-chemical barrier to stop pathogen penetration (Malinovsky et al., 2014; Höfte and Voxeur, 2017). Indeed, plants are constantly challenged by a wide range of pathogens. Many plant cell wall properties and defence mechanisms have been investigated by generating transgenic plants that either silence or over-express cell wall-related genes; see review by Bacete et al. (2018) with a focus on *Arabidopsis thaliana*. These genetic studies have investigated the role of cell wall alterations during pathogen infection as well as defence stimulation by elicitors. The use of screening systems by measuring plant hormone levels, callose deposition, lignin and other biochemical markers is common. Such an approach was used to show that the *A. thaliana* leucine-rich repeat receptor kinase MIK2 was involved in sensing biotic and abiotic stressors and, in so doing, activating stress response pathways (Van der Does et al., 2017). Immunofluorescence staining has also been used to compare cell wall compositional changes during infection with root-knot nematodes, focusing on changes in cell wall polysaccharides observed for *Arabidopsis*, maize and Azuki bean plants (Bozbuga et al., 2018). A range of transcriptomic, proteomic and metabolomic approaches have also been employed to decipher plant-pathogen interactions and identify pathogen and/or plant effectors that are essential for virulence (Plett and Martin, 2018). From a molecular perspective identifying the regulatory network is important; as an example, the transcription factor WRKY33 was shown to be important in activating defence pathways against *Botrytis cinerea* in *A. thaliana* (Sham et al., 2017).

*B. cinerea* (grey rot) is one of the most serious plant pathogens, as it occurs worldwide and can infect more than 1000 different species of host plants (Elad et al., 2016). Recent studies suggest that *B. cinerea* might be fairly ubiquitous, but asymptomatic in many organisms, or during most of the hosts life cycle, waiting for favourable conditions in the form of an endophyte (Sowley et al., 2009; van Kan et al., 2014). In addition to its necrotrophic nature, *B. cinerea* seems to have a short-lived biotrophic stage during early infection on hosts (roughly the first 36 hours post-infection, before a lesion spreads, as described by (Eizner et al., 2017; Veloso and van Kan, 2018).

The capacity of *B. cinerea* to infect the host organism depends on the molecular dialogue between the pathogen and plant. For example, a knockout mutant for the BcExo70 subunit of the exocyst complex, greatly impacted fungal development and pathogenicity (Guan et al., 2019). *B. cinerea* also secretes numerous secondary metabolites involved in pathogenicity such as terpenes, abscisic acid, carotenoids and polyketides, this includes botrydial and botcinic acid which have been recently highlighted (Collado and Viaud, 2016). Some *Botrytis* species do display a narrow host range (Valero-Jiménez et al., 2019), but *B. cinerea* is a very wide-ranging adaptive pathogen. Comparative study on wild and domesticated tomato using 97 different *B. cinerea* isolates (genetically different) showed the fungal capacity to develop different infection strategies, with a highly polygenic virulence response, shown with single-nucleotide polymorphisms analysis, which suggested that the fungi customise their virulence level depending on the host (Breeze, 2019; Soltis et al., 2019).

Fungal pathogens have developed a range of strategies to breach the cell wall. Many fungi are opportunistic and penetrate host plants through stomata and/or wounds. Some develop an appressorium which creates physical pressure differentials in order to breach the cell wall (Howard and Valent, 1996; Mendgen and Hahn, 2002; Ryder and Talbot, 2015). Necrotrophic fungi develop an appressorium-like structure that further secretes cell wall degrading enzymes (CWDEs) into the plant cell wall regions. These CWDEs break down the cell wall polysaccharides in order to penetrate and colonise their host plant (Prins et al., 2000; van Kan, 2006). In the apoplast, fungal effectors can have a dual action, for example, fungal enzymes possess their primary enzymatic functions but have also been shown to trigger plant defence responses such as apoptosis as is the case for BcXYG1, a xyloglucanase (Zhu et al., 2017). When becoming pathogenic, *B. cinerea* has to breach the plant tissue in order to develop necrotic lesions. In order to degrade the cell wall, a wide range of pectinolytic enzymes are secreted, these include endo-polygalacturonases (ePG), pectate lyases and pectin methyl esterases (Tudzynski and Kokkelink, 2009). The ePGs, in particular, have been shown to be essential for *B. cinerea* virulence (Have et al., 1998; Wubben et al., 2000; ten Have et al., 2001, 2002; Kars et al., 2005). They hydrolyse the de-esterified polygalacturonic acid (PGA) stretches of the homogalacturonan (HG) network, one of the major pectin components of the cell wall (Jayani et al., 2005). The pectinolytic fungal enzyme action that degrades the host plant cell wall has been shown to release oligogalacturonides (OG) of various lengths into the apoplast. Oligogalacturonides participate in the plant pathogen dialogue, they are perceived as damage-associated molecular patterns (DAMP) that trigger the stimulation of defence response mechanisms including the hypersensitive response (HR) with the secretion of reactive oxygen species (ROS) or the production of pathogenesis-related proteins (Esquerré-Tugayé et al., 2000; Vorwerk et al., 2004; Ferrari et al., 2013; Benedetti et al., 2015). The HR is an efficient response against biotrophic fungi as it kills plant cells around the infection area to limit its development, but the same response benefits the necrotrophic fungi which have been shown to trigger or enhance it (Govrin and Levine, 2000; Rossi et al., 2017). Necrotrophic pathogens, such as *B. cinerea*, can feed and develop on dead tissue and so are not impeded, but rather aided by the HR response during infection.

Early detection of the pathogen to trigger the plant defence system has been shown to be crucial for effective resistance responses. Recognition of pathogen-associated molecular patterns, conserved molecules from the pathogen and DAMPs, degradation products endogenous to the plant (Gust et al., 2017; Saijo et al., 2018), are recognised by pattern recognition receptors and lead to the activation of the pattern-triggered immunity (Heil and Land, 2014; De Lorenzo et al., 2018; Saijo and Loo, 2019). Pattern recognition receptors are mostly receptor kinases, plasma membrane proteins with an extracellular receptor domain and a cytoplasmic kinase domain, which induce a signal phosphorylation cascade that leads to the activation of plant defence genes (Trdá et al., 2015; Li et al., 2016; Wolf, 2017). Pattern-triggered immunity, in contrast, is a non-specific defence response, commonly associated with callose deposition, lignification and production of ROS (Toruño et al., 2016). There is also effector-triggered immunity, which involves intracellular receptors that recognise pathogen effectors and then trigger a strong response (e.g. HR) at the infection zone area and systemically to other parts of the plant (Cui et al., 2015; Peng et al., 2018). Much of the plant-pathogen communication at the infection site is mediated by hormones such as jasmonic acid and salicylic acid which leads to differential gene expression pathways in the host and fungal systems respectively (Zhang et al., 2017).

To protect the plant against CWDEs, plants can reorganise their cell wall matrix to make it more resistant to degradation. As an example, inactivation of the *reduced wall acetylation 2* gene in *A. thaliana* leads to ca. 20% decrease in cell wall polysaccharide acetylation compared to the wild type, with the mutant plant showing increased resistance to *B. cinerea* (Manabe et al., 2011). In contrast, mutation of the endogenous *A. thaliana* pectin methylesterase inhibitor genes *pmei10*, *pmei11*, and *pmei12*, which encode proteins that regulate pectin methylesterification, have been linked to increased susceptibility to pathogenic fungi (Lionetti et al., 2017). To inhibit fungal ePGs, some plants produce polygalacturonase-inhibiting proteins (PGIPs) (De Lorenzo and Ferrari, 2002; Gomathi and Gnanamanickam, 2004). Polygalacturonase-inhibiting proteins are conserved leucine-rich repeat proteins (Di Matteo et al., 2003, 2006) that are able to inhibit fungal ePG activity (Joubert et al., 2007; Liu et al., 2017). Site-directed mutagenesis studies (Federici et al., 2001; Benedetti et al., 2013) and work on PGIP2 from *Phaseolus vulgaris* against PG1 from *Fusarium moniliform* (Sicilia et al., 2005) showed a mixed mode of inhibition occurring. Furthermore, a PGIP from *P. vulgaris* displayed the ability to bind to pectin and limit ePG access (Spadoni et al., 2006). Polygalacturonase-inhibiting proteins have also been shown to stimulate the production of OGs that can then act as DAMPs triggering plant defence responses downstream (D’Ovidio et al., 2004; Benedetti et al., 2015; Davidsson et al., 2017; Del Corpo et al., 2024).

Characterisation of the *Vvi-PGIP1* isolated from grapevine (*Vitis vinifera*) has indicated multiple functions and molecular roles in disease resistance. Expression of the grapevine PGIP1 in tobacco plants results in enhanced resistance against *B. cinerea* infection (Joubert et al., 2006). Transgenic PGIP1 tobacco plants display altered hormonal levels compared to wild-type plants (Alexandersson et al., 2011; Basson, 2017). The PGIP1 plants also displayed upregulated cinnamyl alcohol dehydrogenase activity with increased lignin staining of stems and downregulation of XET/XTH enzyme levels in leaves (Alexandersson et al., 2011; Joubert et al., 2013). The *VviPGIP1* transgenic plants most notably have an altered arabinoxyloglucan content which is correlated with a tighter, more resistant leaf cell wall composition (Nguema-Ona et al., 2013a). The *VviPGIP1* plants, known to have enhanced resistance to *Botrytis* infection, will serve as a model patho-system with *B. cinerea* as a pathogen to screen plant cell wall degradation and/or reorganisation during fungal infection. In this study, two *VviPGIP1* tobacco lines were subjected to whole plant infections with *B. cinerea* (grape strain) and followed over a 72-hour time course. In addition to visual inspection, scanning electron microscopy was used to characterise the infection progression, comparing wild type to transgenic lines. Furthermore, leaf cell wall composition was also performed using comprehensive microarray polymer profiling (CoMPP) and monosaccharide profiles using gas chromatography coupled mass spectrometry (GC-MS). This was performed on local lesion sites as well as on adjacent tissue (to the lesion) of the infected leaf as well as on systemic uninfected leaves on each of the experimental plants. This surveying approach appeared most suitable to ‘track’ the changes in polymer abundance and turnover during the *B. cinerea* infection progression.

## 2 Materials and methods

### 2.1 Plant material and growth conditions

Plant material consisted of the control tobacco (wild-type *Nicotiana tabacum cv Petit Havana* SR1) and two *VviPGIP1* transgenic lines, namely 37 and 45 (see Joubert et al. (2006) for more details). Seeds were germinated on Murashige and Skoog media (Murashige and Skoog, 1962) with (transgenic) and without (SR1) kanamycin selection. Seedlings were first grown in peat plugs (Jiffy Products International AS, Norway) and then transferred to pots with a final mixture of soil, peat moss and vermiculite. The growth room temperature was kept at 25 °C under artificial lights with a 16-hour light to 8-hour dark photoperiod. Organic fertilizer (Nitrosol, Fleuron (Pty) Ltd, South Africa) was added during watering once every two weeks. Plant infection experiments were conducted on plants with six fully expanded leaves. The first leaf, L1, is the smallest, close to the shoot apical meristem and the L6, is the oldest fully expanded leaf.

### 2.2 *Botrytis cinerea* sporulation and culturing

*B. cinerea* spores previously isolated from a South African vineyard and the strain described as hypervirulent in (Joubert et al., 2006) were used for infection experiments. *B. cinerea* stock cultures were grown on sterile apricot halves (Naturlite, Tiger Food Brands Limited, South Africa) and kept in the dark for two weeks at 23 °C to induce sporulation. Spores were harvested with sterile water and filtrated through glass wool-filled tips to remove the hyphae and other cell debris. Prior to infection, the spores were hydrated overnight in water at 4 °C, and their concentration was calculated with a haemocytometer. Spore viability was assessed by placing a few drops of spore mixtures on a 1% (w/v) water agar plate and calculating the percentage germination after 12 h at 23 °C.

### 2.3 Plant infection experiments

Fully expanded tobacco plants were transferred to infection chambers 24 hours before the start of the infection to acclimate them (as before at 25 °C, under artificial lights with a 16-hour light to 8-hour dark period). However, the chamber was kept at 100% humidity. The day before an infection experiment, spores were harvested as described previously and re-suspended in filtered 50% grape juice solution (Liqui-Fruit Red Grape Juice) at a 1 x 10^6^ spores mL^-1^ concentration. Eight spots of 5 μL (equivalent to a total of 5000 spores) were deposited on the adaxial side of each L3, L4 and L5 tobacco leaf (**Figure 1**). The experiment was conducted according to the validated pathosystem as outlined in Joubert et al. (2006).

**Figure 1:**
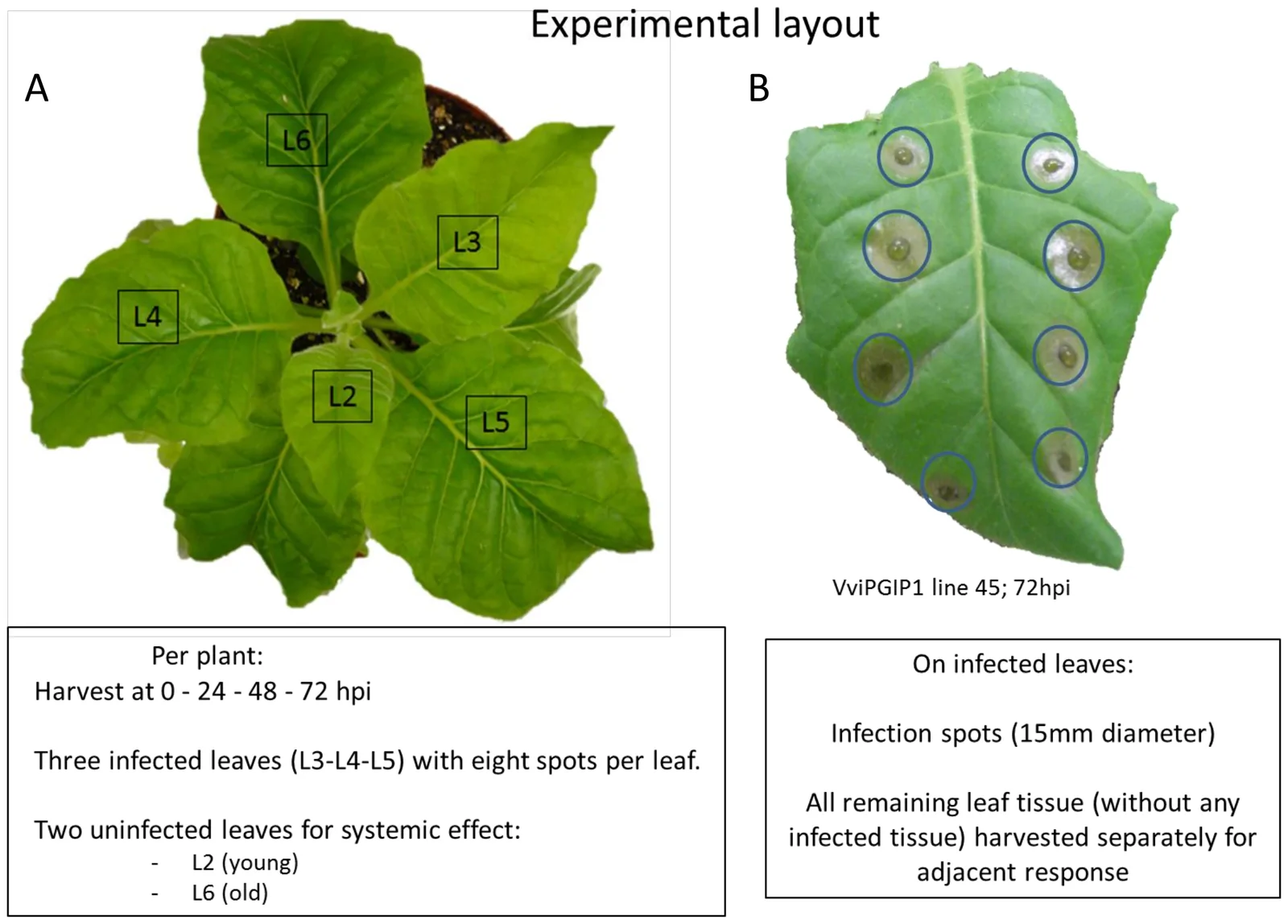
**(A)** Photograph of a typical tobacco plant used for the study to indicate leaf positions harvested (indicated in the black boxes). Out of the eight leaves on the plant at the time of the experiment, leaf 2 was the youngest harvested one, close to the apical meristem and leaves 4–6 fully expanded leaves. Leaves one, seven and eight were not harvested. **(B)** Photograph of an infected leaf 72 hpi. For analysis, tissue inside the blue circles (15 mm diameter) represents the lesions and is harvested separately from the rest of the leaf, which represents adjacent tissue.

### 2.4 Sampling during the infection process

To follow the plant infection process, a time course experiment was established, and samples were harvested at different times: 0, 24, 48 and 72 hours post-inoculation (hpi). To track local lesion development, samples were generated from leaves L3, L4 and L5. Leaf disks, 15 mm in diameter around the infection spots, were isolated using a cork borer and processed separately from the remaining leaf tissue. The remaining infected L3, L4 and L5 leaf tissue were labelled and processed as the ‘adjacent response’ tissue. The uninfected leaves L2 and L6 were also harvested to track distal (systemic) response to the fungus infection. For each line and time point, samples from four individual plants (biological repeats) were harvested and their respective disks and leaves pooled together according to their leaf position. Four plants were mock inoculated (using water and grape juice) and harvested at 72 hpi in addition to uninfected plants.

### 2.5 Scanning electron microscopy

Infection progression was tracked using a Leo^®^ 1430VP Scanning Electron Microscope (Central Analytical Facility, Stellenbosch University). Images were taken at 24, 48, 72, and 96 hpi with *B. cinerea* spore suspension. Prior to imaging, leaf tissue was cut and carefully mounted on aluminium stubs, centring on the infection spots, using double-sided carbon tape for adherence. Beam conditions during imaging were 7 kV at approximately 1.5 nA resolution.

### 2.6 Cell wall isolation protocol

Alcohol insoluble residue (AIR) was prepared from tobacco leaves as outlined in (Nguema-Ona et al., 2012). Leaf material was frozen using liquid nitrogen and ground to a powder with a Retsch Mixer Mill (Retsch, Haan, Germany) set at 30 rounds per minute for 60 seconds. The powder was boiled in 80% aqueous ethanol for 20 minutes. Insoluble material was washed with methanol for two hours on a rotating wheel. The pellets were sequentially washed twice with a (1:1) methanol/chloroform solution for two hours then rinsed with acetone for another two hours and air dried. The extracted material was re-suspended in water and freeze-dried to obtain cell wall AIR which was used as material for all further analytical experiments. All solvents used were from (Sigma-Aldrich, MO, USA) at reagent grade.

### 2.7 Gas chromatography-mass spectrometry for monosaccharides

Monosaccharide analysis using GC-MS was performed according to (York et al., 1986) with some modifications. Hydrolysis of AIR was performed using 2 M TFA incubated at 110 °C for two hours. Sequential derivatization was performed first at 80 °C for 16 h using 1 M methanolic HCl and thereafter, 20 min with an HMDS + TMCS + Pyridine (3:1:9) (Sylon HTP) kit (Sigma-Aldrich). Silylation was performed at 80 °C. Trimethyl-silyl-glycosides were then dissolved in cyclohexane prior to injection into the GC-MS system. A gas chromatograph Agilent 6890 N (Agilent, CA, USA) coupled to an Agilent 5975 MS mass spectrometer detector was used for analysis. A polar (95% dimethylpolysiloxane) ZB-Semivolatiles Guardian (30 m, 0.25 mm ID, 0.25 µm film thickness) part number 7HG-G027–11 GC column was used. Arabinose, rhamnose, fucose, xylose, mannose, galacturonic acid, galactose, glucose and glucuronic acid were quantified using standards (from Sigma-Aldrich, MO, USA). The software Xcalibur (Thermo Fisher Scientific Inc., MA, USA) was used for integration and quantitation. Results are expressed in mg g^-1^ of AIR, and statistical significance was calculated with an ANOVA (P = 0.05).

### 2.8 Comprehensive microarray polymer profiling for polysaccharides-proteins

Comprehensive microarray polymer profiling (see Moller et al. (2007)) on tobacco AIR was performed as described in Nguema-Ona et al. (2012). 300 µL of CDTA (diamino-cyclohexane-tetra-acetic acid), which mainly extracts pectins, was added to 10 mg of AIR and shaken for two hours at 60 °C. 300 µL NaOH (4 M + 0.1% NaBH4), which mainly extracts hemicelluloses, was added to the pellet and shaken for another two hours. CDTA and NaOH fractions were printed onto nitrocellulose membranes (Whatman, pore size 0.45 mm Whatman, Maidstone, UK) using a microarray printer (Sprint, Arrayjet, Roslin, UK), with two replicates and four dilutions each per sample. A set of monoclonal antibodies (mAbs) and carbohydrate-binding modules (CBMs) were used to probe the printed arrays (see **Table 1** for the detailed list of antibodies and associated references). Arrays were developed and quantified using a flatbed scanner, and results were presented as semi-quantitative heat maps. The highest value was set at 100, and all other values were normalised accordingly. A cut-off of five was applied at the lower end.

**Table 1:** List of monoclonal antibodies and carbohydrate-binding modules used for the comprehensive microarray polymer profiling analysis.

Homogalacturonan		low DE	JIM5	Knox et al., 1990; Clausen et al., 2003
		high DE	JIM7	
		Partially ME	LM18	Verhertbruggen et al., 2009a
		Partially ME	LM19	
		Partially ME	LM20	
		HG in “egg-box” motif Xylogalacturonan	2F4 LM8	Liners et al., 1989 Willats et al., 2004
RG-I associated		Backbone of RG-I	INRA-RU1	Ralet et al., 2010
		Backbone of RG-I	INRA-RU2	
		D-galactan	LM5	Jones et al., 1997
		L-arabinan	LM6	Willats et al., 1998
		Linearised L-arabinan	LM13	Verhertbruggen et al., 2009b
	Mannan	(galacto)(gluco)mannan	LM21	Marcus et al., 2010
		D-(gluco)mannan	LM22	
	Glucan	β-D-glucan	BS-400-2	Meikle et al., 1991
	Xyloglucan	Xyloglucan backbone (XXXG motif)	LM15	Marcus et al., 2008
		Xyloglucan	LM25	Pedersen et al., 2012
	Xylan	β-D-xylan	LM10	McCartney et al., 2005
		β-D-xylan/arabinoxylan	LM11	
Cellulose		Cellulose (crystalline)	CBM3a	Tormo et al., 1996
Proteins	Extensins		LM1	Smallwood et al., 1995
			JIM11	Smallwood et al., 1994 Knox et al., 1995
			JIM20	
	AGP		JIM8	McCabe et al., 1997
			JIM13	Knox et al., 1991; Yates et al., 1996
			LM14	Moller et al., 2008
		β-linked GlcA	LM2	Smallwood et al., 1996

### 2.9 Statistical and multivariate data analysis

Each sample represented four leaves at the same developmental stage, taken from different plants and pooled together before processing. Thus, for all experiments, at least 12 plants were tested for each genotype. The changes observed in the data obtained from the various analysis were analysed for statistical significance using univariate statistical analysis software tools (Statistica 10, Statsoft Southern Africa Analytics, Sandton, South Africa). Multivariate analysis, such as principal component analysis, was performed with the SIMCA 14 software package (Sartorium Stedim Biotech - Umetrics AB, Umea, Sweden).

## 3 Results

### 3.1 *Botrytis cinerea* infection and lesion development of wild type versus *VviPGIP1* tobacco

Infections were carried out using *B. cinerea* (grape strain) spores placed on wild type versus *VviPGIP1* tobacco lines and assessed visually for lesion development according to a defined pathosystem. A lesion index developed by Carstens et al. (2003) was used as a baseline comparative reference. In summary, the wild-type tobacco *N. tabacum cv Petit Havana* (SR1) was selected as control with two transgenic tobacco lines (lines 37 and 45) expressing *VviPGIP1*, which were shown to have an increased resistance to *B. cinerea*. These results replicated observations described in Joubert et al. (2006) showing a resistance phenotype for the transgenic tobacco expressing *VviPGIP1*, clearly visible from 72–96 hpi onwards.

At the 24 hpi stage, the tissue beneath the infection droplet spot retained a healthy appearance, although the first signs of light browning and primary lesion development were also evident (**Figures 2A–C**). Later, at 48 hpi, the primary lesion had begun to spread away from the infection spore-filled droplet and signs of tissue maceration, indicative of type 2 and/or type 3 lesions (**Figures 2D–F**), were present with no difference between genotypes. After 72 hpi, the lesions had enlarged with two different zones visible; an inner dark, wet and macerated zone where the spore drop was placed, surrounded by a light brown and dry ring (**Figures 2G–I**). For some of the infected tissues, a thin, darker ring was visible at the interface with the non-infected leaf area. Chlorosis was also visible on the “healthy” leaf tissue around the infection area. However, at this stage, no difference was evident between wild-type and transgenic leaves with regard to symptom progression. At 96 hpi, differences started to become noticeable between PGIP lines and the SR1 plants. At 96 hpi in SR1, the lesion had spread beyond the second ring forming a third ring of macerated tissue, looking similar to the primary infection area and characterised as a type 6 lesion (**Figure 2J**). However, for the PGIP transgenic lines 37 and 45, the infection spread had ceased (**Figures 2K, L**) and remained similar to the lesions present at 72 hpi showing evidence of infection ‘containment’ (**Figures 2H, L**). At 192 hpi or five days later, infected SR1 tobacco displayed lesions that had spread throughout the leaf and tissue maceration was widespread (**Figure 2M**). Leaf shedding in heavily infected plants was also observed (F. Weiller, personal observation). In contrast for PGIP transgenic lines 37 and 45, a reduced and limited area remained infected (**Figures 2N, O**) on the leaves. For the PGIP lines, the primary lesions around the infection spot were clearly visible with macerated tissue. Two infection rings were present: one clear and dry of two to three millimetres in diameter, and the second, darker, of about one millimetre in diameter, dry and of a consistency close to non-infected tissue (not yet macerated). Based on a lesion index from Carstens et al. (2003), these correspond to type 3 lesions. Chlorosis was also observed as a whitening of the tissue surrounding the infected area (**Figure 2O**). Less damage, i.e. lesion size and colour, was observed for PGIP transgenic line 37 compared to 45, suggesting a slightly enhanced resistance phenotype, as previously observed by Joubert et al. (2006).

**Figure 2:**
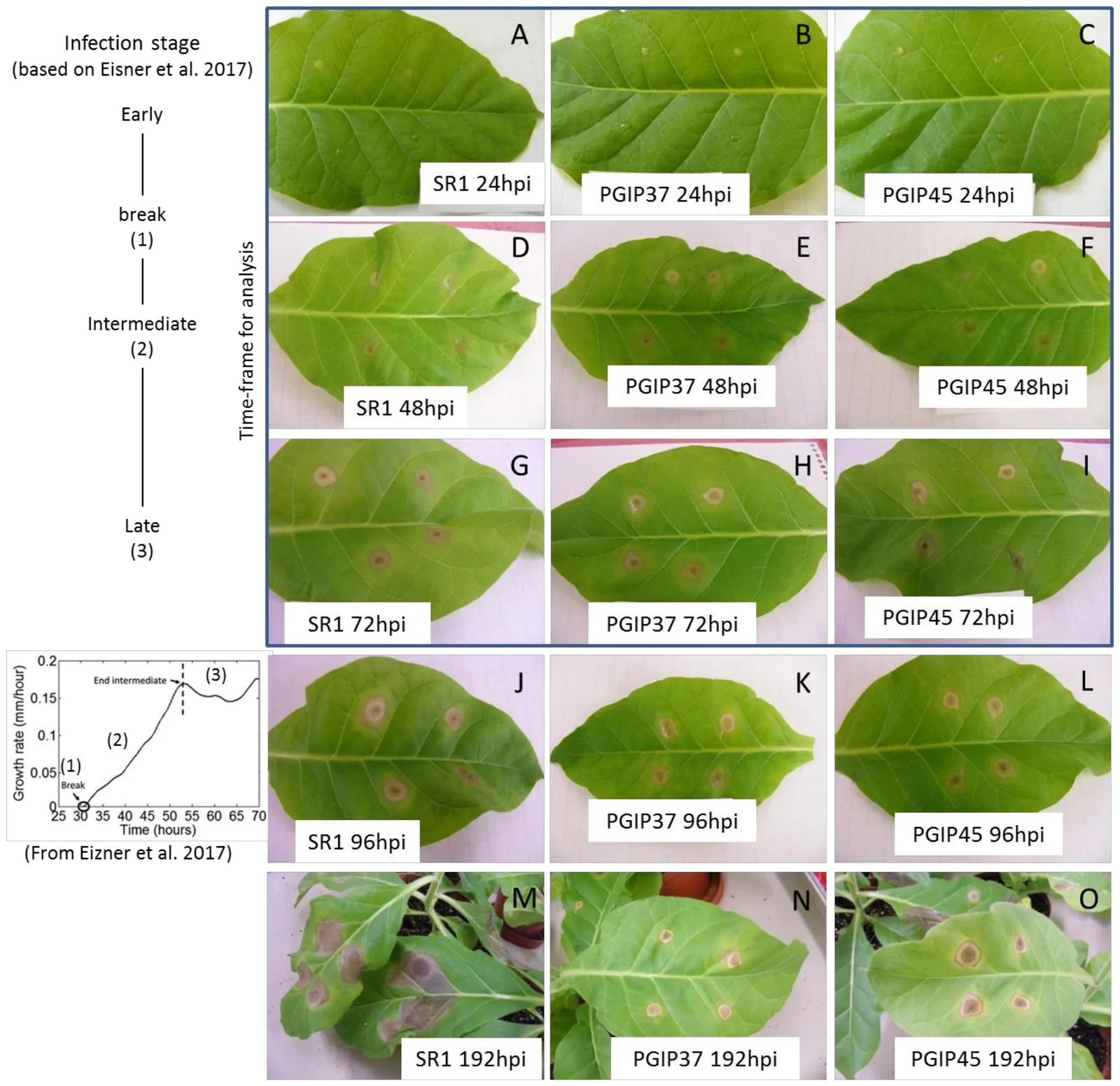
Photographs of representative infected leaves of the wild-type SR1 and the transgenic lines *VviPGIP1* line 37 and line 45 infected with *B. cinerea* over time. The blue frame represents the time frame selected for biochemical analysis based on the infection stages described in Eizner et al. (2017) as shown on the left side. Photographs are labelled **(A-O)** indicating hpi (hours post infection) and for cross-referencing with text.

Scanning electron microscopy was used to track infection progression under high magnification levels. The infected tobacco leaves from the different lines were observed at 24 hpi and 72 hpi. As PGIP lines 37 and 45 produced similar images, only line 37 are shown here in comparison to SR1 tobacco. Non-infected regions of the SR1 leaf surface show evidence of undamaged cells, stomata and leaf hairs (trichomes) (**Figure 3A**). At a lower magnification, non-infected PGIP line 37 (**Figure 3E**) shows similar undamaged tissue with dark circles indicative of microdroplets of water present at the leaf surface. Images of a 24 hpi lesion at two different magnification levels for SR1 (**Figures 3B, C**) and for PGIP line 37 (**Figures 3F, G**) are shown. The entire lesion after 24 hpi in SR1 is presented in **Figure 3B**, subsequent imaging at the centre (**Figure 3C**) reveals the presence of multiple holes indicative of tissue maceration. The centre zone would be where the *B. cinerea* spores were deposited. In the same way for PGIP line 37, the lesion after 24 hpi is presented in **Figure 3F**; interestingly here, evidence of collapsed leaf hairs beyond the lesion zone was noted. **Figure 3E** reveals high-magnified images of the lesion centre where, again, multiple holes are present, revealing tissue maceration and collapse. After 72 hpi (**Figures 3D, H**), fungal structures were visible at the leaf surface in the infection zone, with a network of hyphae on the necrotised tissue for both wild type and PGIP line.

**Figure 3:**
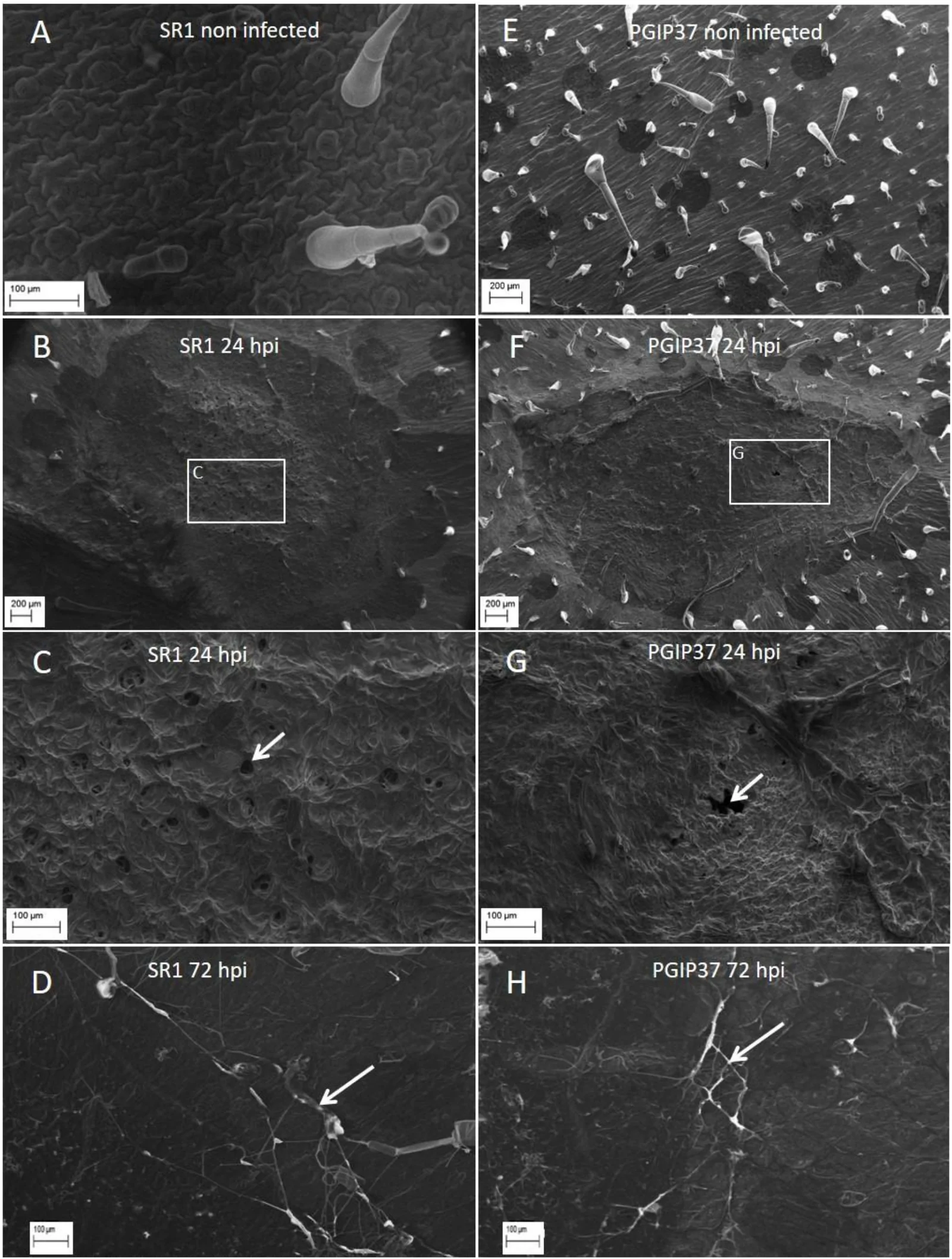
Representative micrographs of the surface of infected tobacco leaves were observed with a scanning electron microscope. **(A–D)** SR1; **(E–H)** *VviPGIP1* line 37; The arrows show tissue degradation on **(C)** and **(G)** and fungal hyphae on **(D)** and **(H)**; **(A)**, **(E)**: non-infected SR1 and *VviPGIP1* plant leaf surface used as a control.

In general, no difference was noted in lesion ultrastructure between the wild-type and the transgenic lines PGIP lines 37 and 45 in the first 72 hpi. Non-infected leaf tissue of the three lines was also observed with a transmission electron microscope, but no difference could be seen between any of the lines (data not shown).

### 3.2 Monosaccharide compositional analysis using GC-MS of lesions and adjacent tissue

To evaluate broad changes in cell wall composition during the first 72 hpi, AIR samples from lesions were hydrolysed, and their monosaccharide components were analysed using GC-MS (**Table 2**). The monosaccharide profiles of the three genotypes were very similar. An increase in GalA level with leaf age (position) was observed for both wild-type and transgenic plants. A marked and significant decrease in GalA from 0 hpi to 72 hpi was mirrored by an inverse increase in Glc for all three genotypes at the lesion site. For the lesion tissue, a decrease for Xyl and Gal was observed in the first 24 hpi and a slight increase in Man at 48 hpi for both wild-type and transgenic plants (**Table 2**).

**Table 2:** Monosaccharide composition of the cell wall, expressed in mg g^-1^ of the wild-type SR1, PGIP37 and PGIP45 tobacco leaf AIR of infected tissue (lesion).

	Time (hpi)	Ara	Rham	Fuc	Xyl	Man	Gal	GalA	Glc	GlucA
SR1	0	13.45 ± 0.8^ab^	12.51 ± 0.73^a^	2.55 ± 0.84^a^	17.31 ± 0.52^c^	6.05 ± 0.92^abcd^	24.16 ± 0.73^c^	88.41 ± 0.53^c^	41.02 ± 10.38^a^	5.09 ± 0.7^ab^
SR1	24	10.9 ± 1.47^a^	11.8 ± 0.83^a^	2.57 ± 0.36^a^	9.68 ± 1.42^a^	4.66 ± 0.97^a^	13.91 ± 1.87^a^	43.99 ± 8.29^a^	47.85 ± 6.97^a^	4.19 ± 0.46^a^
SR1	48	16.41 ± 2.41^ab^	12.35 ± 1.83^a^	2.45 ± 0.12^a^	10.87 ± 1.63^ab^	6.62 ± 1.55^abcd^	15.59 ± 2.19^ab^	32.09 ± 7.61^a^	37.11 ± 37.24^a^	6.3 ± 1.25^b^
SR1	72	17.69 ± 1.11^b^	13.05 ± 0.85^a^	2.24 ± 0.11^a^	11.45 ± 0.68^ab^	10.03 ± 2.06^d^	16.38 ± 0.91^ab^	45.79 ± 22.25^ab^	48.48 ± 30.35^a^	5.92 ± 0.36^ab^
PGIP45	0	10.2 ± 1.53^a^	11.28 ± 1.68^a^	2.19 ± 0.24^a^	14.17 ± 1.97^bc^	5.13 ± 0.59^abc^	19.96 ± 2.69^bc^	80.42 ± 13.06^bc^	19.54 ± 6.23^a^	4.55 ± 0.69^ab^
PGIP45	24	11.87 ± 2.25^ab^	12.42 ± 2.06^a^	2.22 ± 0.16^a^	9.76 ± 2.27^a^	3.41 ± 1.47^a^	14.07 ± 3.05^a^	45 ± 14.25^ab^	32.62 ± 5.29^a^	4.4 ± 0.13^ab^
PGIP45	48	15.62 ± 2.24^ab^	12.48 ± 0.25^a^	2.69 ± 1.12^a^	10.42 ± 0.29^ab^	6.76 ± 0.55^abcd^	14.92 ± 0.42^ab^	42.96 ± 5.73^a^	46.56 ± 5.02^a^	4.71 ± 0.45^ab^
PGIP45	72	15.54 ± 0.3^ab^	12.42 ± 1.24^a^	2.01 ± 0.09^a^	10.71 ± 0.72^ab^	9.42 ± 1.13^bc^d	15.39 ± 0.98^ab^	29.99 ± 5.02^a^	68.19 ± 2.58^a^	5.23 ± 0.4^ab^
PGIP37	0	12.77 ± 2.93^a^	13 ± 0.55^a^	2.38 ± 0.78^a^	16.97 ± 0.7^a^	5.35 ± 1.03^a^	23.74 ± 0.95^a^	64.79 ± 15.47^ab^	20.23 ± 8^ab^	4.34 ± 0.55^a^
PGIP37	24	12.32 ± 1.94^a^	12.84 ± 0.65^a^	1.9 ± 0.32^a^	15.44 ± 2.19^a^	5.29 ± 0.79^a^	21.7 ± 2.9^a^	70.91 ± 5.1^ab^	20.65 ± 7.11^ab^	4.67 ± 0.11^a^
PGIP37	48	10.75 ± 0.75^a^	12.23 ± 0.15^a^	2.25 ± 0.61^a^	15.5 ± 0.76^a^	5.71 ± 0.72^a^	21.76 ± 1.04^a^	60.68 ± 6.49^ab^	42.51 ± 10.88^ab^	4.21 ± 0.12^a^
PGIP37	72	11.66 ± 2.3^a^	11.34 ± 1.35^a^	3.33 ± 0.37^a^	14.4 ± 2.75^a^	4.93 ± 1.14^a^	20.24 ± 3.72^a^	50.63 ± 4.79^a^	35.51 ± 15.54^ab^	4.38 ± 0.4^a^

The standard deviation represents the average of four biological repeats. hpi, hours post inoculation; Ara, arabinose; Rha, rhamnose; Fuc, fucose; Xyl, xylose; Man, mannose; Gal, galactose; GalA, galacturonic acid; Glc, glucose; GlcA, glucuronic acid. The std dev displays the significant letters showing the different statistical groups after an ANOVA (P = 0.05).

### 3.3 Comprehensive microarray polymer profiling of wild-type tobacco (SR1) infected with *Botrytis cinerea* (as a baseline)

Comprehensive microarray polymer profiling analysis uses a wide variety of mAbs and CBMs which bind to various pectin, hemicellulose and cell wall protein epitopes. Leaf developmental patterns for cell wall probes were evident in the CoMPP dataset (**Figure 4**). A general increase in signal for mAbs binding to HG with leaf age was evident in the datasets for adjacent and distal tissue of the CDTA fraction (**Figure 4**). 2F4, for example, a mAb that binds to calcium ion egg box motifs present in pectins, followed this increasing trend with leaf age. A decreasing signal for mAbs LM5 and LM6 (galactans and arabinans) that form part of RG-I (rhamnogalacturonan I) side chains was observed. mAbs INRA-RU1 and INRA-RU2, which bind the RG-I backbone, showed a relatively invariant signal pattern. Probes also recognised extensin (mAbs LM1, JIM11, JIM20) and AGP (arabinogalactan protein) (mAbs JIM8, JIM13, LM2) epitopes in the leaf tissue. Differences in adjacent and distal tissue to the infection were slight, and the overall patterning was similar to the ontogenic leaf age effect (**Figure 4**).

**Figure 4:**
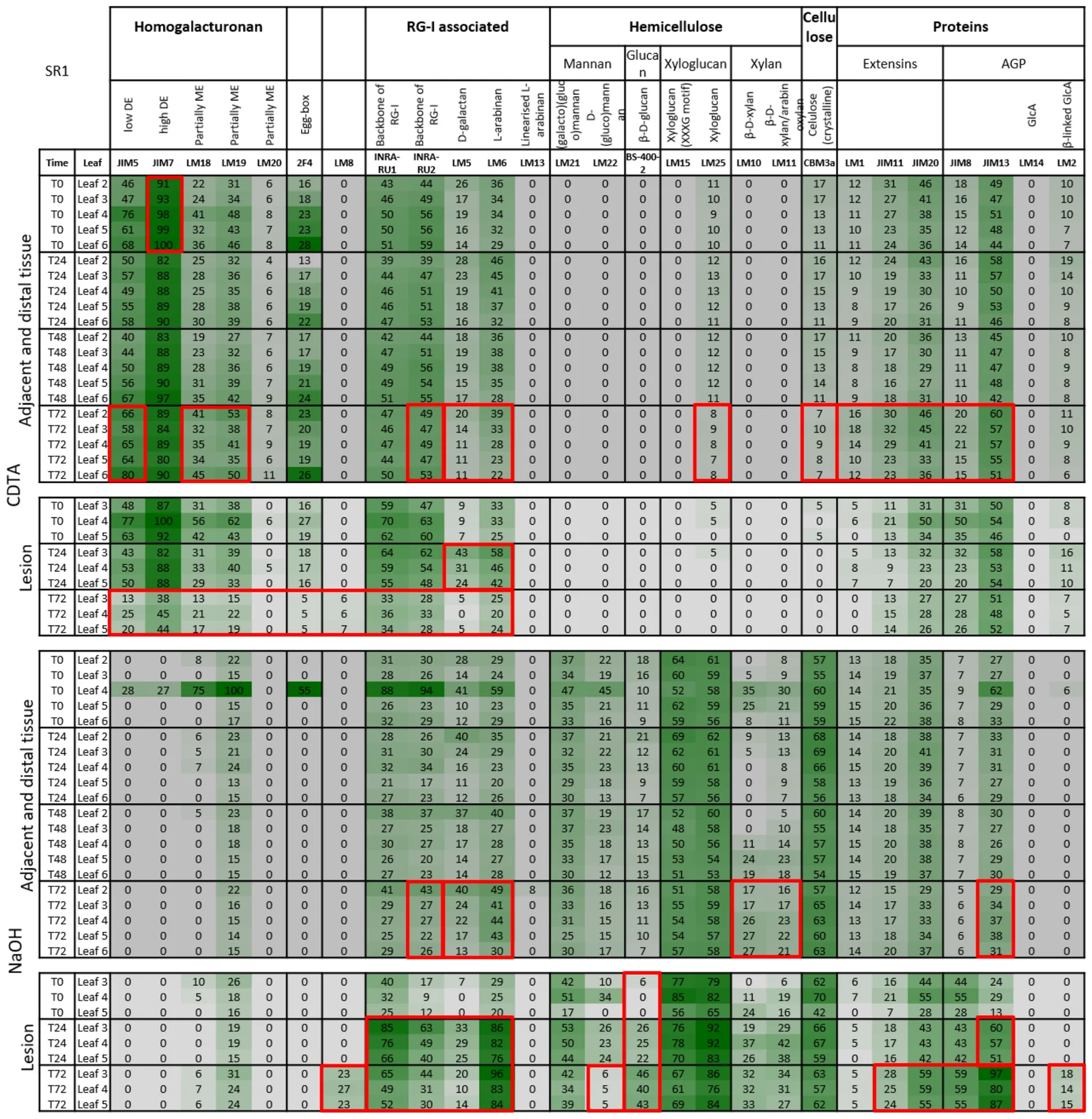
Comprehensive microarray polymer profiling analysis of the wild-type SR1, tobacco leaf AIR infected by *B. cinerea*. Results are presented as heatmaps of both CDTA and NaOH extractions, showing the relative abundance of cell wall associated epitopes and separated between non-infected tissue (adjacent and distal to the infection) and infected tissue (lesion). A detailed list of the antibodies used is available in **Table 1**.

At the lesion site (**Figure 4**) however, major changes in epitope abundance for HG, RG-I, AGP and extensin epitopes were noticed in the CDTA but mainly in the NaOH fraction. From 0 hpi to 24 hpi there was an increase in signal for mAbs LM5 and LM6 (arabinans and galactans) in the CDTA fraction. Thereafter the signals decreased, suggesting de-pectination/degradation of the epitopes in the CDTA component. This would support pectin degradation occurring due to pathogen infection. Homogalacturonan epitopes (mAbs JIM5, JIM7, LM18, LM19) and RG-I backbone (INRA-RU1, INRA-RU2) signals decreased at 72 hpi supporting evidence of signal degradation. In the CDTA fractions of the lesions, epitopes associated with AGPs (mAbs JIM11, JIM20) and extensins (mAbs JIM8, JIM13) remained relatively unchanged. In the NaOH fractions of the lesions, marked changes were evident at 24 hpi and 72 hpi. INRA-RU1, INRA-RU2, LM5 and LM6 all increased after 24 hpi suggesting pectin exposure of underlying RG-I networks of the cell wall. LM22 glucomannan increased at 24 hpi and then decreased to 72 hpi in the NaOH fraction. Glucans (recognised by BS-400-2) increased stepwise at 24 hpi and 72 hpi in the alkali extract. Xyloglucans recognised by mAbs LM15 and LM25 followed a decreasing or increasing then decreasing signal in the datasets. Interestingly, the LM8 probe, which binds to xylogalacturonan (XGA), was detected at 72 hpi in the NaOH-soluble material. AGP and extensin probes JIM11, JIM20, JIM8 and JIM13 all increased markedly at 24 hpi and 72 hpi in the lesion tissue.

### 3.4 Comprehensive microarray polymer profiling of SR1, PGIP line 37 and PGIP line 45 infected with *Botrytis cinerea*

The previous analysis characterised *B. cinerea* infection progression on SR1 tobacco plants. To understand the influence of cell wall modifications and PGIP1 expression on fungal resistance in the transgenic lines, a further study was undertaken. It was decided to focus on leaf 4 in the comparative analysis (to minimise the ontogenic impacts, and at this stage, the leaf is fully expanded and mature. Additional data for leaf 3 and leaf 5 are available as **Supplementary Datasets** (**Supplementary Figures S1**-**S6**).

At the lesion site, evidence of pectin degradation was present following the 0 hpi, 24 hpi and 72 hpi time points for all three genotypes (**Figure 5**). Homogalacturonan-associated mAbs JIM5, JIM7, LM18, LM19 and 2F4 all decreased from 0 hpi to 72 hpi in the CDTA extract. Similarly, for RG-I associated mAbs INRA-RU1, INRA-RU2, LM5 and LM6, the probe signals decreased from 24 hpi onwards. The extensin probes JIM11 and JIM20, and the AGP probes JIM8 and JIM13 decreased at 24 hpi. No major differences between wild-type and transgenic material were present. In the case of the NaOH extract RG1 probes (INRA-RU1, INRA-RU2, LM5 and LM6), increased at 24 hpi and remained high at 72 hpi. Mannans (LM21 and LM22) decreased with infection progression. Xyloglucans detected by mAbs LM15 and LM25 as well as CBM3a followed a decreasing trend in the datasets. Extensins (JIM11 and JIM20) and AGPs (JIM8 and JIM13) showed a general trend of increasing in abundance from 24 hpi to 72 hpi. The AGP LM2 epitope was detectable at 72 hpi, and the LM8 probe for xylogalacturonan was present at 72 hpi for all lines. Again, no major differences were observed between SR1 and the PGIP1 expressing lines at the infection lesion over the first 72 hpi.

**Figure 5:**
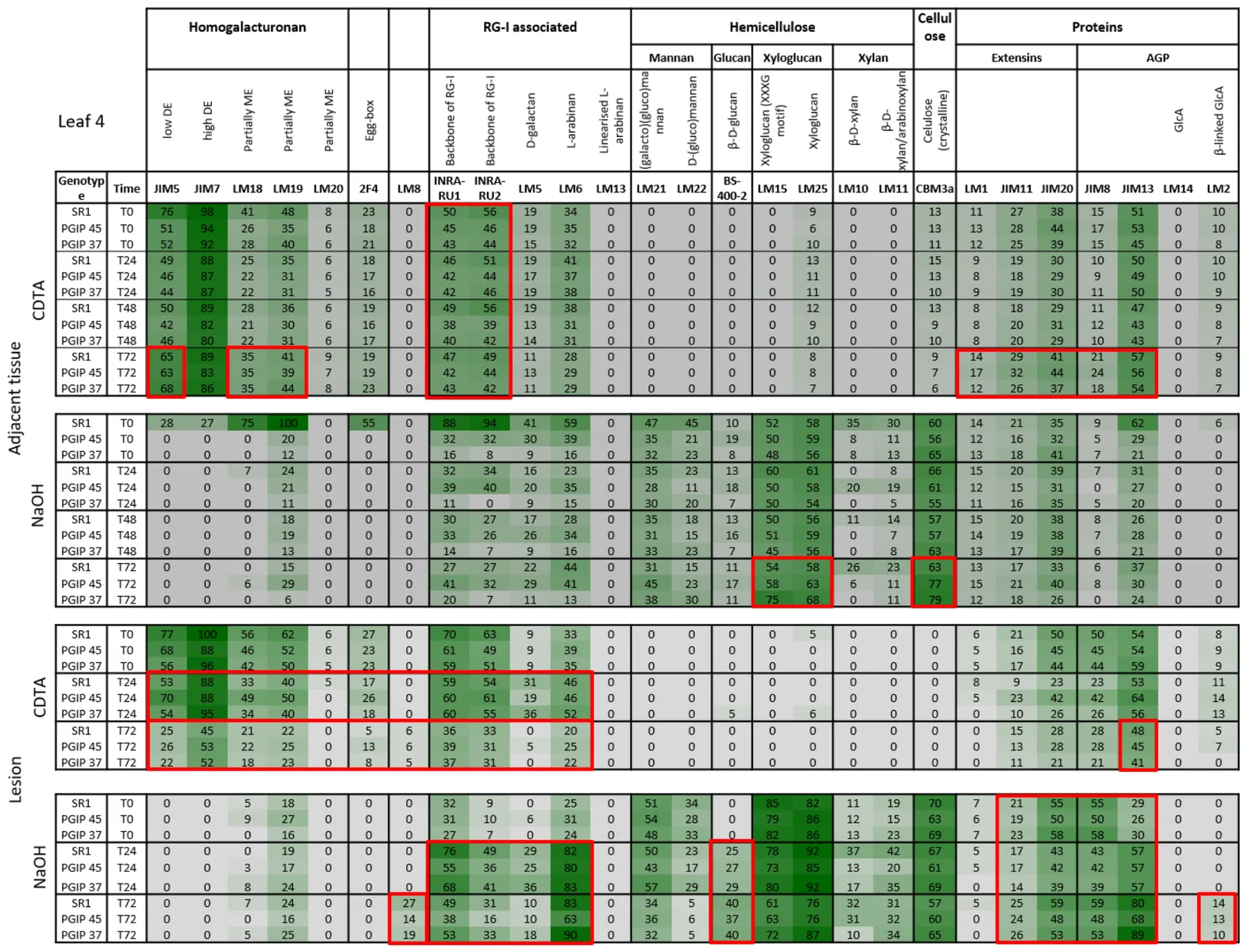
Comprehensive microarray polymer profiling analysis of the wild-type SR1, PGIP37 and PGIP45 AIR of the leaf four lesion and adjacent tissue to the lesion. Results are presented as heatmaps showing the relative abundance of cell wall-associated epitopes (see material and methods for more information). A detailed list of the antibodies used is available in **Table 1**.

Adjacent and distal tissue were also evaluated using CoMPP arrays (**Figures 5**, **6**; **Supplementary Figures S1**-**S6**). For adjacent tissue, the major patterns in the CDTA datasets show JIM5, JIM7, LM18, LM19 and 2F4 as the major signals along with RG-I backbones (INRA-RU1 and INRA-RU2) and side chains (LM5 and LM6) (**Figure 5**). Extensins (LM1, JIM11 and JIM20) and AGPs (JIM8 and JIM13) also present in CDTA extracts increased in abundance in all lines at 72 hpi as a general response to infection. For the NaOH extracts, the major RG-I backbones (INRA-RU1 and INRA-RU2) and side chains (LM5 and LM6) showed a decreasing signal, especially for PGIP line 37 for all time points. Interestingly, LM21 and LM22, which bind to mannans, showed an increasing trend for PGIP lines 37 and 45 compared to the WT. LM15, LM25 and CBM3a signals from xyloglucans were present, and these increased markedly for PGIP line 37 at 72 hpi compared to the wild type. NaOH soluble extensin (mAbs LM1, JIM11, JIM20) and AGP (mAbs JIM8 and JIM13) epitopes remained relatively unchanged over time. For possible impacts on distal (systemic) tissues, analysis was conducted on leaf 2 (L2 – youngest leaf) and leaf 6 (L6 – oldest leaf) (**Figure 6**). The CDTA extract of L2 and L6 showed signals for HG (mAbs JIM5, JIM7, LM18, LM19), RG-I (mAbs INRA-RU1, INRA-RU2, LM5, LM6), extensins (mAbs LM1, JIM11, JIM20) and AGPs (JIM8, JIM13, LM2). A maturation pattern between L2 and L6 can be seen, as LM5 (galactans), LM6 (arabinans) and LM2 (AGPs) decreased from L2 to L6, whereas pectin egg-boxes increased (mAb 2F4), however no clear differences between SR1 and transgenic PGIP1 line 37 and 45 were evident. For the NaOH extract, the major signals were for RG-I (mAbs INRA-RU1, INRA-RU2, LM5, LM6), mannans (mAb LM21, LM22), xyloglucan (mAbs LM15, LM25, CBM3a), extensins (LM1, JIM11 and JIM20) and AGPs (JIM8 and JIM13). Here differences for mannans (mAb LM21, LM22), xyloglucan (mAbs LM15, LM25, CBM3a) and xylans (mAbs LM10, LM11) between wild-type and transgenic lines were observed. PCA plots of the CoMPP arrays are provided in the heatmaps to visualize the data-structures (See **Supplementary Materials**).

**Figure 6:**
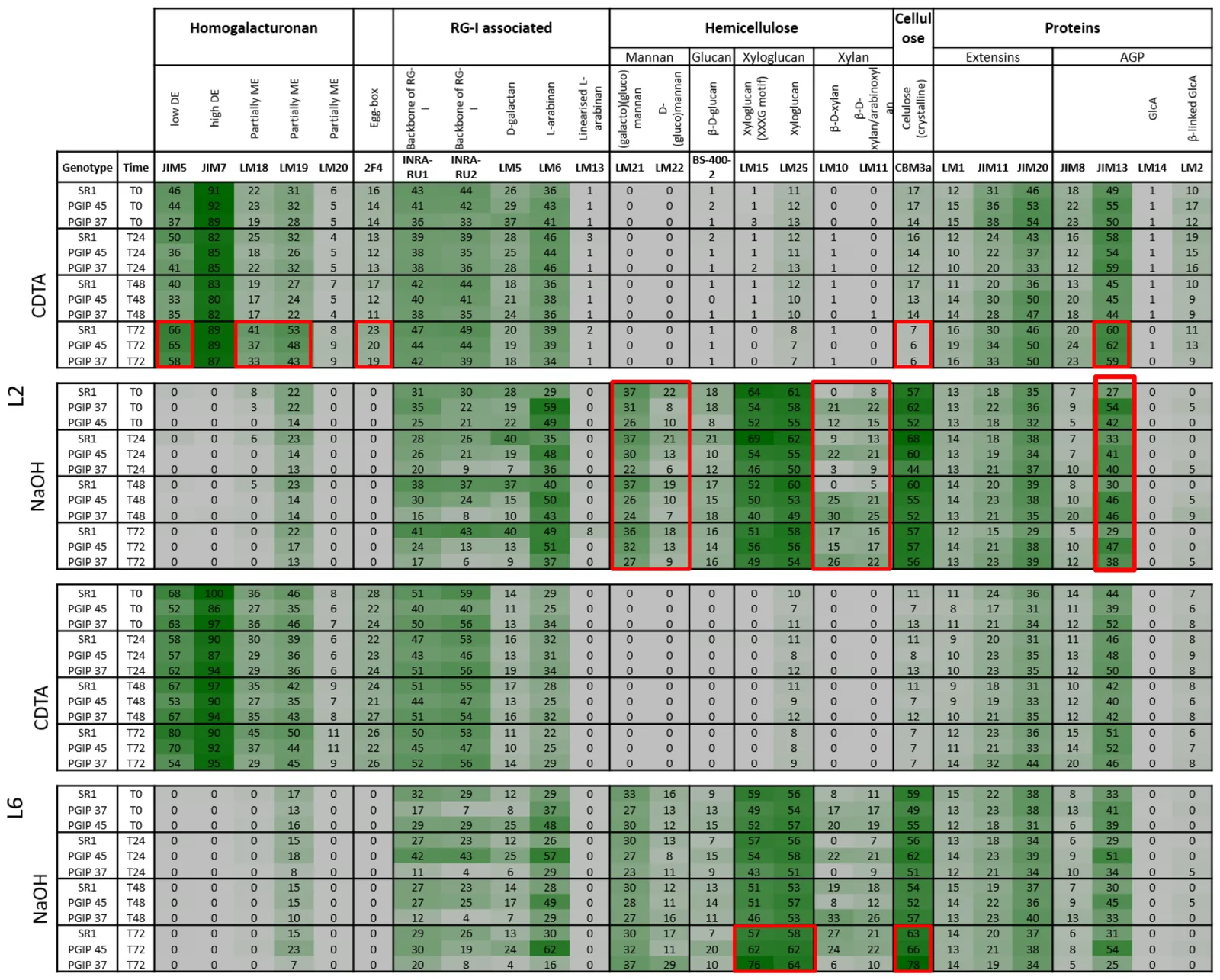
Comprehensive microarray polymer profiling analysis of the wild-type SR1, PGIP37 and PGIP45 tobacco leave AIR of distal tissue to the infection (non-infected leaves L2 and L6). Results are presented as heatmaps showing the relative abundance of cell wall-associated epitopes from the CDTA and NaOH extractions for both L2 and L6 leaves (see material and methods for more information). A detailed list of the antibodies used is available in **Table 1**.

## 4 Discussion

### 4.1 No visual phenotype differences during *Botrytis cinerea* infection, irrespective of PGIP1 expression during the first 72 hpi.

*N. tabacum* cv. *Petit Havana* is not an easily susceptible host to the necrotrophic fungus *B. cinerea* as it accumulates numerous alkaloids such as scopoletin, essential oils and lipids, phenylpropanoids and PR proteins in response to fungal infection (El Oirdi et al., 2010). Infection on this host can however be promoted with a high concentration of spores under favourable conditions of temperature and humidity (Hain et al., 1993; Joubert et al., 2006). Overexpression of Vvi-PGIP1 in tobacco has led to enhanced resistance to the necrotrophic *B. cinerea* compared to wild-type plants (Joubert et al., 2006). Our experiments have shown that the first 72 hpi appears crucial for effective plant resistance to be mounted, particularly in the areas surrounding the infection spot and in systemic tissues. The infection progression slowed down and was contained after 72 hpi in the transgenic plants compared to wild-type plants. The 72 hpi period corresponded to the lag phase of infection (intermediate phase), which preceded the late phase (after 96 hpi) of fungal infection outlined by (Eizner et al., 2017). *B. cinerea* first penetrates the leaf tissue which corresponds with the multiple holes visible in the lesion visible during the early stages of infection. After tissue penetration, it grows into a mycelium network beneath the epidermis and starts to degrade the tissue inducing necrosis. At 72 and 96 hpi we observed external (ectopic) mycelium formation on the necrotised tissue close to the border of the infection similar to what was described previously in tomato by (Prins et al., 2000). Electron microscopic observations on tomato and broad beans showed that the *B. cinerea* spores germinated and grew an appressorium-like structure that enzymatically breaks through the cuticle and cell wall layers (Rijkenberg et al., 1980; Mansfield and Richardson, 1981). *B. cinerea* often enters a quiescent stage, after penetration into the host, producing a symptomless infection at early stages (Prins et al., 2000; Emmanuel et al., 2018; Veloso and van Kan, 2018) and later developing an expanding lesion with tissue maceration (Elad, 1988; Prins et al., 2000; van Kan, 2005). That wasn’t the case in this study, where all inoculation spots developed into a lesion, except in the cases where the initial droplet had evaporated before the formation of the lesion. *B. cinerea* can develop a variety of different kinds of local lesions (Carstens et al., 2003). In addition, it should be noted that the fungal biomass can contribute to the glucose and mannose datasets presented however this was not tested for explicitly.

In our experimental conditions (high spore concentration, high humidity and favourable temperature), *B. cinerea* had optimal conditions to develop a successful lesion, but in the transgenic plants, a thin dark and dry ring was formed between healthy and macerated tissue at 72 and 96 hpi. Shlezinger et al. (2011) have shown that the first 48 hpi are crucial for *B. cinerea.* It was also shown in transgenic tobacco expressing a grapevine phytoalexin, resveratrol, that its rapid accumulation at the local lesion site in the first 48 hpi was correlated with enhanced plant resistance to pathogenic fungi (Hain et al., 1993). Veloso and van Kan (2018) showed that the ability of *B. cinerea* to successfully infect plants depended on its capacity to shut down plant autophagy early in favour of apoptosis. Basson (2017) followed transcriptional changes of *N. tabacum* during the first 48 hpi after *B. cinerea* infection. The study showed that *VviPGIP1* transgenic tobacco shut down photosynthesis much faster than the wild type, moving from source to sink metabolism. Transgenic plants also regulated their oxidative burst differently by producing less hydrogen peroxide, similar to a recent finding in *A. thaliana* with *Gh*PGIP1 (Liu et al., 2017). Production of ROS is essential in the plant HR against biotrophic fungi, but necrotrophic fungi like *Botrytis* use it to their advantage to infect the tissue. The observations that PGIPs can influence ROS levels in addition to their role in plant cell wall functioning suggests that these proteins have multi-functional roles in plant immunity and plant defence. Linked to our observations, this suggests that resistant plants can quickly create a buffer zone surrounding the macerated tissue and confine the fungi to stop their expansion.

### 4.2 *Botrytis cinerea* degrades pectin-rich leaf cell walls in transgenic and wild-type tobacco

*B. cinerea* life cycle and infection process have been widely described, and numerous virulence factors have been characterised (van Kan, 2005, 2006; Williamson et al., 2007; Shah et al., 2012). The CWDEs have been shown to be crucial for *B. cinerea* virulence and aid in its capacity to breach the plant cuticle and cell wall layers (Have et al., 1998; ten Have et al., 2002; Tudzynski and Kokkelink, 2009). Our results showed a general depectination via degradation of the HGs in the infected tobacco tissue with a strong decrease of signal for mAbs such as JIM7 and a drop in GalA levels at the local lesion site. GalA is found in the cell wall as PGA chains forming the HG pectin backbone. Polygalacturonase-inhibiting protein 1 has well documented anti-ePG activity (Esquerré-Tugayé et al., 2000; D’Ovidio et al., 2004; Sicilia et al., 2005; Di Matteo et al., 2006; Joubert et al., 2007; Kalunke et al., 2015; Li and Smigocki, 2018) in addition to its capacity to bind pectin and prevent HG degradation (Spadoni et al., 2006). Homogalacturonan degradation is mediated by CWDEs, which also release mono-sugars which can be used for fungal growth (Blanco-Ulate et al., 2015). Polygalacturonic acid degradation, for example, has been shown to be essential for *Aspergillus niger* growth as shown in Alazi et al. (2016). Short PGAs termed OGs are essential for plant protection as they act as DAMPs to signal infection and trigger plant immune responses (Boutrot and Zipfel, 2017; Bacete et al., 2018). It is also known that OGs length influence the strength and duration of the defence response (Davidsson et al., 2017; Hou et al., 2019). Polygalacturonase-inhibiting proteins have been shown to promote the production of OGs (Benedetti et al., 2015). Despite its signalling role participating in defence mechanisms induction, HG was observed as more susceptible to matrix disorganisation and maceration progression. Indeed, RG-I polymers appeared much more resistant to fungal degradation than HG epitopes. *B. cinerea* appears to be more limited in releasing RG-I targeting enzymes; only RG acetyl-esterase, for example, has been reported (Blanco-Ulate et al., 2014). RG-I cross-links exist between xyloglucan and xylan (Popper and Fry, 2005, 2008; Ralet et al., 2016; Broxterman and Schols, 2018). Associations have also been shown between xyloglucan and cellulose as well as pectin (Zykwinska et al., 2007; Park and Cosgrove, 2015). The presence of co-polymers with pectins may impede degradation by fungal enzymes during the experimental time-frames of this study. Basson (2017), in a transcriptional study of *VviPGIP1* tobacco plants under *B. cinerea* infection, showed that at 48 hpi, some defence-related genes involved in defence-associated proteins or secondary metabolites biosynthesis were starting to be expressed in leaves distal to the infection. This response is likely to have been mediated by jasmonic acid, rather than salicylic acid signalling (Benedetti et al., 2015; Liu et al., 2017). From a cell wall compositional point of view, polymer variations were observed from 72 hpi. Adjacent tissue had elevated levels of HGs and xyloglucan/cellulose. Distal leaves followed an ontogenic pattern response to the infection, with younger leaves having higher HG values [with various methylesterification levels as observed in fruit; see Kelloniemi et al. (2015)] and older leaves with elevated xyloglucan/cellulose. Considering the variations of the cell wall matrix during leaf maturation, plants seem to adapt their response to the infection accordingly. In that regard, the *VviPGIP1* plants showed a distinct response with increased levels of xyloglucan/cellulose in adjacent tissue. This stronger signal observed for xyloglucan in the transgenic plants is probably linked to the downregulation of XET/XTH observed in the non-infected plants (Alexandersson et al., 2011).

### 4.3 RG-I and AGP-extensin proteins are deposited in response to *Botrytis cinerea* in transgenic and wild-type tobacco

Cell wall proteins have established roles in plant defence described and reviewed by Showalter (1993); Cassab (1998) and Deepak et al. (2010). Hydroxy-proline-rich glycoproteins (HRGP) are a family of cell wall proteins which function in plant defence in addition to multiple other functions that are being characterised (Deepak et al., 2010; Rashid, 2016). Extensin gene expression is induced in *A*. *thaliana* during infection with *Xanthomonas campestris* and *Pseudomonas syringae* (Merkouropoulos and Shirsat, 2003; Wei and Shirsat, 2006). In roots, extensin epitopes have been linked to infection by *Fusarium oxysporum* of banana (Wu et al., 2017). Similarly, extensin upregulation is found in *Solanum tuberosum* when infected by *Pectobacterium atrosepticum* (Koroney et al., 2016). In addition, *A. thaliana* mutants impaired in extensin arabinosylation were found to have increased susceptibility to *Phytophthora parasitica* (Castilleux et al., 2020). The role of extensins in plant pathosystems was recently reviewed in (Castilleux et al., 2018). In our study Vvi-PGIP1 overexpression could thus be mediating extensin deposition and act as a priming response. In a similar manner to extensins; AGPs are HRGPs with multiple functions in plant growth and development (Showalter, 2001). AGPs have roles in plant defence against biotic and abiotic stressors; *in vitro* studies have shown their capacity to attract zoospores and inhibit *Aphanomyces euteiches* growth and development (Cannesan et al., 2012). In roots, they have been shown to be very important for rhizobium interactions (reviewed by Nguema-Ona et al. (2013b)). In bananas, AGP expression and distribution are stimulated by *F. oxysporum* infection (Wu et al., 2017). In *Oryza sativa*, infection with *Magnaporthe oryzae* induces increased expression of AGPs, suggesting a role in plant-pathogen signalling (Liang et al., 2018). In *N. alata* Na*-*AGP4 expression was found to be modulated by *B. cinerea* presence (Gilson et al., 2001) and additional roles for AGPs in pathogen interactions have been summarised in Mareri et al. (2018). It is therefore interesting to observe the accumulation of AGPs in the lesion area after *B. cinerea* infection or their elevated levels in adjacent tissue and distal leaves after 72 hpi. However, given the limited timeframe of the study window, 72 hpi, additional research investigations are warranted.

### 4.4 Conclusion

This study followed cell wall modifications during an infection by *B. cinerea*, between sensitive and resistant plants to the fungi. It was conducted using a classical combination of microscopy and monosaccharide quantification completed with the recently developed glycan microarray profiling technique (CoMPP).

The overexpression of Vvi-PGIP1 has been shown to induce cell wall modifications prior to infection, and this work is, to our knowledge, the first report to present an overview of the cell wall transformations during fungal infection. The most notable effect observed was the depectination with HG degradation and RG-I exposure. During the timeframe of our experiment, hemicellulose, including the xyloglucan/cellulose network, showed limited degradation, especially in the PGIP plants. A differential response was observed between the lesion, adjacent and distal tissue with an accumulation of glycoproteins at and around the infection site. This is interesting as the role of glycoproteins, namely extensins and AGPs, in plant defence is not fully understood. Extensin has been described as a defence protein, but its mode of action is only starting to be deciphered in roots. As for AGP, the topic is very new and direct evidence of its protective role is still lacking.

The compositional role of the cell wall in plant immunity is also suggested with the accumulation of polysaccharides and proteins at the lesion site. It should be noted fungal biomass such as glucans and mannans also accumulate at the lesion. These changes were not limited to the infection area but started to be detectable in distal tissue and non-infected leaves, signs of a systemic response to the fungal attack. Despite susceptibility differences to *B. cinerea* between transgenic and control plants relatively minor cell wall compositional changes, it might be the timing of response that matters. The authors are aware however that we lack additional time-points of infection and integration of transcriptomic datasets that could lead to stronger causal arguments being advanced.

Together with the unpublished transcriptomic datasets these results show that the response to *B. cinerea* infection is multifactorial and includes various mechanisms such as a quick switch of the infected organ from source to sink, a modulation of the HR (less ROS production), the strengthening of the cell wall and the production and accumulation of defence proteins. The *VviPGIP1* tobacco – *B. cinerea* patho-system has helped elucidate the role of PGIP in the defence and plant response to infection. The present work presented suggests a clear role for grapevine PGIP1 in modifying cell wall composition *in planta* to protect against *Botrytis cinerea* infection.

## Data Availability

The original contributions presented in the study are included in the article/**Supplementary Material**. Further inquiries can be directed to the corresponding author.

## References

[B1] Alazi E. Niu J. Kowalczyk J. E. Peng M. Aguilar Pontes M. V. van Kan J. A. L. . (2016). The transcriptional activator GaaR of *Aspergillus niger* is required for release and utilization of d-galacturonic acid from pectin. FEBS Lett. 590, 1804–1815. doi: 10.1002/1873-3468.12211 27174630 PMC5111758

[B2] Alexandersson E. Becker J. V. W. Jacobson D. Nguema-Ona E. Steyn C. Denby K. J. . (2011). Constitutive expression of a grapevine polygalacturonase-inhibiting protein affects gene expression and cell wall properties in uninfected tobacco. BMC Res. Notes 4, 493. doi: 10.1186/1756-0500-4-493 22078230 PMC3339426

[B3] Bacete L. Mélida H. Miedes E. Molina A. (2018). Plant cell wall-mediated immunity: cell wall changes trigger disease resistance responses. Plant J. 93, 614–636. doi: 10.1111/tpj.13807 29266460

[B4] Basson C. E. (2017). Transcriptomic analysis of disease resistance responses using a tobacco-*Botrytis cinerea* pathosystem ( Stellenbosch University, Stellenbosch).

[B5] Benedetti M. Andreani F. Leggio C. Galantini L. Di Matteo A. Pavel N. V. . (2013). A single amino-acid substitution allows endo-polygalacturonase of *Fusarium verticillioides* to acquire recognition by PGIP2 from *Phaseolus vulgaris*. PLoS One 8, e80610. doi: 10.1371/journal.pone.0080610 24260434 PMC3834070

[B6] Benedetti M. Pontiggia D. Raggi S. Cheng Z. Scaloni F. Ferrari S. . (2015). Plant immunity triggered by engineered *in vivo* release of oligogalacturonides, damage-associated molecular patterns. PNAS 112, 5533–5538. doi: 10.1073/pnas.1504154112 25870275 PMC4418913

[B7] Blanco-Ulate B. Labavitch J. M. Vincenti E. Powell A. L. T. Cantu D. (2015). “ Hitting the wall: Plant cell walls during *Botrytis cinerea* infections,” in Botrytis – the Fungus, the Pathogen and its Management in Agricultural Systems (Cham: Springer Nature), 361–386. doi: 10.1007/978-3-319-23371-0_18

[B8] Blanco-Ulate B. Morales-Cruz A. Amrine K. C. H. Labavitch J. M. Powell A. L. T. Cantu D. (2014). Genome-wide transcriptional profiling of *Botrytis cinerea* genes targeting plant cell walls during infections of different hosts. Front. Plant Sci. 5. doi: 10.3389/fpls.2014.00435 25232357 PMC4153048

[B9] Boutrot F. Zipfel C. (2017). Function, discovery, and exploitation of plant pattern recognition receptors for broad-spectrum disease resistance. Annu. Rev. Phytopathol. 55, 257–286. doi: 10.1146/annurev-phyto-080614-120106 28617654

[B10] Bozbuga R. Lilley C. J. Knox J. P. Urwin P. E. (2018). Host-specific signatures of the cell wall changes induced by the plant parasitic nematode, *Meloidogyne incognita*. Sci. Rep. 8, 17302. doi: 10.1038/s41598-018-35529-7 30470775 PMC6251906

[B11] Breeze E. (2019). 97 shades of gray: Genetic interactions of the gray mold, *Botrytis cinerea*, with wild and domesticated tomato. Plant Cell 31, 280–281. doi: 10.1105/tpc.19.00030 30651347 PMC6447008

[B12] Broxterman S. E. Schols H. A. (2018). Characterisation of pectin-xylan complexes in tomato primary plant cell walls. Carbohydr. Polym. 197, 269–276. doi: 10.1016/J.CARBPOL.2018.06.003 30007613

[B13] Cannesan M. A. Durand C. Burel C. Gangneux C. Lerouge P. Ishii T. . (2012). Effect of arabinogalactan proteins from the root caps of pea and *Brassica napus* on *Aphanomyces euteiches* zoospore chemotaxis and germination. Plant Physiol. 159, 1658–1670. doi: 10.1104/pp.112.198507 22645070 PMC3425204

[B14] Carstens M. Vivier M. A. Pretorius I. S. (2003). The *Saccharomyces cerevisiae* chitinase, encoded by the CTS1-2 gene, confers antifungal activity against *Botrytis cinerea* to transgenic tobacco. Transgenic Res. 12, 497–508. doi: 10.1023/A:1024220023057 12885170

[B15] Cassab G. I. (1998). Plant cell wall proteins. Annu. Rev. Plant Physiol. Plant Mol. Biol. 49, 281–309. doi: 10.1146/annurev.arplant.49.1.281 15012236

[B16] Castilleux R. Plancot B. Gügi B. Attard A. Loutelier-Bourhis C. Lefranc B. . (2020). Extensin arabinosylation is involved in root response to elicitors and limits oomycete colonization. Ann. Bot. 125, 751–763. doi: 10.1093/aob/mcz068 31242281 PMC7182588

[B17] Castilleux R. Plancot B. Ropitaux M. Carreras A. Leprince J. Boulogne I. . (2018). Cell wall extensins in root–microbe interactions and root secretions. J. Exp. Bot. 69, 4235–4247. doi: 10.1093/jxb/ery238 29945246

[B18] Clausen M. H. Willats W. G. T. Knox J. P. (2003). Synthetic methyl hexagalacturonate hapten inhibitors of anti-homogalacturonan monoclonal antibodies LM7, JIM5 and JIM7. Carbohydr. Res. 338, 1797–1800. doi: 10.1016/S0008-6215(03)00272-6 12892947

[B19] Collado I. G. Viaud M. (2016). “ Secondary metabolism in Botrytis cinerea: Combining genomic and metabolomic approaches,” in Botrytis – the Fungus, the Pathogen and its Management in Agricultural Systems ( Springer International Publishing, Cham), 291–313. doi: 10.1007/978-3-319-23371-0_15

[B20] Cui H. Tsuda K. Parker J. E. (2015). Effector-triggered immunity: From pathogen perception to robust defense. Annu. Rev. Plant Biol. 66, 487–511. doi: 10.1146/annurev-arplant-050213-040012 25494461

[B21] D’Ovidio R. Mattei B. Roberti S. Bellincampi D. (2004). Polygalacturonases, polygalacturonase-inhibiting proteins and pectic oligomers in plant-pathogen interactions. Biochim. Biophys. Acta - Proteins Proteomics 1696, 237–244. doi: 10.1016/j.bbapap.2003.08.012 14871664

[B22] Davidsson P. Broberg M. Kariola T. Sipari N. Pirhonen M. Palva E. T. (2017). Short oligogalacturonides induce pathogen resistance-associated gene expression in *Arabidopsis thaliana*. BMC Plant Biol. 17, 19. doi: 10.1186/s12870-016-0959-1 28103793 PMC5248502

[B23] Deepak S. Shailasree S. Kini R. K. Muck A. Mithöfer A. Shetty S. H. (2010). Hydroxyproline-rich glycoproteins and plant defence. J. Phytopathol. 158, 585–593. doi: 10.1111/j.1439-0434.2010.01669.x40046247

[B24] Del Corpo D. Coculo D. Greco M. De Lorenzo G. Lionetti V. (2024). Pull the fuzes: Processing protein precursors to generate apoplastic danger signals for triggering plant immunity. Plant Commun. 5, 100931. doi: 10.1016/j.xplc.2024.100931 38689495 PMC11371470

[B25] De Lorenzo G. Ferrari S. (2002). Polygalacturonase-inhibiting proteins in defense against phytopathogenic fungi. Curr. Opin. Plant Biol. 5, 295–299. doi: 10.1016/S1369-5266(02)00271-6 12179962

[B26] De Lorenzo G. Ferrari S. Cervone F. Okun E. (2018). Extracellular DAMPs in plants and mammals: Immunity, tissue damage and repair. Trends Immunol. 39, 937–950. doi: 10.1016/j.it.2018.09.006 30293747

[B27] Di Matteo A. Bonivento D. Tsernoglou D. Federici L. Cervone F. (2006). Polygalacturonase-inhibiting protein (PGIP) in plant defence: a structural view. Phytochemistry 67, 528–533. doi: 10.1016/j.phytochem.2005.12.025 16458942

[B28] Di Matteo A. Federici L. Mattei B. Salvi G. Johnson K. A. Savino C. . (2003). The crystal structure of polygalacturonase-inhibiting protein (PGIP), a leucine-rich repeat protein involved in plant defense. Proc. Natl. Acad. Sci. United States America 100, 10124–10128. doi: 10.1073/pnas.1733690100 PMC187787 12904578

[B29] Eizner E. Ronen M. Gur Y. Gavish A. Zhu W. Sharon A. (2017). Characterization of Botrytis-plant interactions using PathTrack © - an automated system for dynamic analysis of disease development. Mol. Plant Pathol. 18, 503–512. doi: 10.1111/joms.12099 PMC6638221 27061637

[B30] Elad Y. (1988). Scanning electron microscopy of parasitism of *Botrytis cinerea* on flowers and fruits of cucumber. Trans. Br. Mycological Soc. 91, 185–190. doi: 10.1016/S0007-1536(88)80025-1

[B31] Elad Y. Pertot I. Cotes Prado A. M. Stewart A. (2016). “ Plant hosts of botrytis spp,” in Botrytis – the Fungus, the Pathogen and its Management in Agricultural Systems ( Springer International Publishing, Cham), 413–486. doi: 10.1007/978-3-319-23371-0_20

[B32] El Oirdi M. Trapani A. Bouarab K. (2010). The nature of tobacco resistance against *Botrytis cinerea* depends on the infection structures of the pathogen. Environ. Microbiol. 12, 239–253. doi: 10.1111/j.1462-2920.2009.02063.x 19799622

[B33] Emmanuel C. J. van Kan J. A. L. Shaw M. W. (2018). Differences in the gene transcription state of *Botrytis cinerea* between necrotic and symptomless infections of lettuce and *Arabidopsis thaliana*. Plant Pathol., 1–9. doi: 10.1111/ppa.12907

[B34] Esquerré-Tugayé M. T. Boudart G. Dumas B. (2000). Cell wall degrading enzymes, inhibitory proteins, and oligosaccharides participate in the molecular dialogue between plants and pathogens. Plant Physiol. Biochem. 38, 157–163.

[B35] Federici L. Caprari C. Mattei B. Savino C. Di Matteo A. De Lorenzo G. . (2001). Structural requirements of endopolygalacturonase for the interaction with PGIP (polygalacturonase- inhibiting protein). Proc. Natl. Acad. Sci. 98, 13425–13430. doi: 10.1073/pnas.231473698 PMC60887 11687632

[B36] Ferrari S. Savatin D. V. Sicilia F. Gramegna G. Cervone F. De Lorenzo G. (2013). Oligogalacturonides: plant damage-associated molecular patterns and regulators of growth and development. Front. Plant Sci. 4, 49. doi: 10.3389/fpls.2013.00049 PMC3595604 23493833

[B37] Gilson P. Gaspar Y. M. Oxley D. Youl J. J. Bacic A. (2001). NaAGP4 is an arabinogalactan protein whose expression is suppressed by wounding and fungal infection in Nicotiana alata. Protoplasma 215, 128–139. doi: 10.1007/BF01280309 11732052

[B38] Gomathi V. Gnanamanickam S. S. (2004). Polygalacturonase-inhibiting proteins in plant defence. Curr. Sci. 87, 1211–1217.

[B39] Govrin E. M. Levine A. (2000). The hypersensitive response facilitates plant infection by the necrotrophic pathogen *Botrytis cinerea*. Curr. Biol. 10, 751–757. doi: 10.1016/S0960-9822(00)00560-1 10898976

[B40] Guan W. Feng J. Wang R. Ma Z. Wang W. Wang K. . (2019). Functional analysis of the exocyst subunit BcExo70 in *Botrytis cinerea*. Curr. Genet. 84. doi: 10.1007/s00294-019-01002-9 31183512

[B41] Gust A. A. Pruitt R. Nürnberger T. (2017). Sensing danger: key to activating plant immunity. Trends Plant Sci. 22, 779–791. doi: 10.1016/J.TPLANTS.2017.07.005 28779900

[B42] Hain R. Reif H.-J. Krause Langebartels R. Kindl H. Vornam B. . (1993). Disease resistance results from foreign phytoalexin expression in a novel plant. Nature 361, 153–156. doi: 10.1016/S1473-3099(02)00353-5 8421520

[B43] Have A. T. Mulder W. Visser J. van Kan J. A. L. (1998). The endopolygalacturonase gene bcpg1 is required for full virulence of *botrytis cinerea*. Mol. Plant-Microbe Interact. 11, 1009–1016. doi: 10.1094/MPMI.1998.11.10.1009 9768518

[B44] Heil M. Land W. G. (2014). Danger signals - damaged-self recognition across the tree of life. Front. Plant Sci. 5, 578. doi: 10.3389/fpls.2014.00578 PMC4215617 25400647

[B45] Höfte H. Voxeur A. (2017). Plant cell walls. Curr. Biology : CB 27, R865–R870. doi: 10.1016/j.cub.2017.05.025 28898654

[B46] Hou S. Liu Z. Shen H. Wu D. (2019). Damage-associated molecular pattern-triggered immunity in plants. Front. Plant Sci. 10 646. doi: 10.3389/fpls.2019.00646 PMC6547358 31191574

[B47] Howard R. J. Valent B. (1996). BREAKING AND ENTERING: host penetration by the fungal rice blast pathogen *Magnaporthe grisea*. Annu. Rev. Microbiol. 50, 491–512. doi: 10.1146/annurev.micro.50.1.491 8905089

[B48] Jayani R. S. Saxena S. Gupta R. (2005). Microbial pectinolytic enzymes: A review. Process Biochem. 40, 2931–2944. doi: 10.1016/J.PROCBIO.2005.03.026

[B49] Jones L. Seymour G. B. Knox J. P. (1997). Localization of pectic galactan in tomato cell walls using a monoclonal antibody specific to (1→4)-[beta]-D-galactan. Plant Physiol. 113, 1405 1412. doi: 10.1104/PP.113.4.1405 PMC158264 12223681

[B50] Joubert D. A. de Lorenzo G. Vivier M. A. (2013). Regulation of the grapevine polygalacturonase-inhibiting protein encoding gene: expression pattern, induction profile and promoter analysis. J. Plant Res. 126, 267–281. doi: 10.1007/s10265-012-0515-5 22932820

[B51] Joubert D. A. Kars I. Wagemakers L. Bergmann C. Kemp G. Vivier M. A. . (2007). A polygalacturonase-inhibiting protein from grapevine reduces the symptoms of the endopolygalacturonase BcPG2 from *Botrytis cinerea* in *Nicotiana benthamiana* leaves without any evidence for *in vitro* interaction. Mol. Plant-Microbe Interact. 20, 392–402. doi: 10.1094/MPMI-20-4-0392 17427809

[B52] Joubert D. A. Slaughter A. R. Kemp G. Becker J. V. W. Krooshof G. H. Bergmann C. . (2006). The grapevine polygalacturonase-inhibiting protein (*VvPGIP1*) reduces *Botrytis cinerea* susceptibility in transgenic tobacco and differentially inhibits fungal polygalacturonases. Transgenic Res. 15, 687–702. doi: 10.1007/s11248-006-9019-1 17072564

[B53] Kalunke R. M. Tundo S. Benedetti M. Cervone F. De Lorenzo G. D’Ovidio R. (2015). An update on polygalacturonase-inhibiting protein (PGIP), a leucine-rich repeat protein that protects crop plants against pathogens. Front. Plant Sci. 6, 146. doi: 10.3389/fpls.2015.00146 PMC4367531 25852708

[B54] Kars I. Krooshof G. H. Wagemakers L. Joosten R. Benen J. A. E. Van Kan J. A. L. (2005). Necrotizing activity of five *Botrytis cinerea* endopolygalacturonases produced in *Pichia pastoris*. Plant J. 43, 213–225. doi: 10.1111/j.1365-313X.2005.02436.x 15998308

[B55] Kelloniemi J. Trouvelot S. Héloir M.-C. Simon A. Dalmais B. Frettinger P. . (2015). Analysis of the molecular dialogue between gray mold (*Botrytis cinerea*) and grapevine (*Vitis vinifera*) reveals a clear shift in defense mechanisms during berry ripening. Mol. Plant-Microbe Interact. 28, 1167–1180. doi: 10.1094/MPMI-02-15-0039-R 26267356

[B56] Knox J. P. Linstead P. J. Cooper J. P. C. Roberts K. (1991). Developmentally regulated epitopes of cell surface arabinogalactan proteins and their relation to root tissue pattern formation. Plant J. 1, 317–326. doi: 10.1046/j.1365-313X.1991.t01-9-00999.x 21736649

[B57] Knox J. P. Linstead P. King J. Cooper C. Roberts K. (1990). Pectin esterification is spatially regulated both within cell walls and between developing tissues of root apices. Planta 181, 512–521. doi: 10.1007/BF00193004 24196931

[B58] Knox J. P. Peart J. Neill S. (1995). Identification of novel cell surface epitopes using a leaf epidermal-strip assay system. Planta 196(2), 266–270. doi: 10.1007/BF0020138330311153

[B59] Koroney A. S. Plasson C. Pawlak B. Sidikou R. Driouich A. Menu-Bouaouiche L. . (2016). Root exudate of *Solanum tuberosum* is enriched in galactose-containing molecules and impacts the growth of *Pectobacterium atrosepticum*. Ann. Bot. 118, 797–808. doi: 10.1093/aob/mcw128 27390353 PMC5055634

[B60] Li H. Smigocki A. C. (2018). Sugar beet polygalacturonase-inhibiting proteins with 11 LRRs confer Rhizoctonia, Fusarium and Botrytis resistance in Nicotiana plants. Physiol. Mol. Plant Pathol. 102, 200–208. doi: 10.1016/J.PMPP.2018.03.00142574925

[B61] Li L. Yu Y. Zhou Z. Zhou J. M. (2016). Plant pattern-recognition receptors controlling innate immunity. Sci. China Life Sci. 59, 878–888. doi: 10.1007/s11427-016-0115-2 27535423

[B62] Liang Y. Zhao J. Wang C. Jing Y. Chengyun L. Baldwin T. C. (2018). Infection with blast fungus (Magnaporthe orzyae) leads to increased expression of an arabinogalactan-protein epitope in both susceptible and resistant rice cultivars. Physiol. Mol. Plant Pathol. 102, 136–143. doi: 10.1016/J.PMPP.2018.01.00242574925

[B63] Liners F. Thibault J. J. Van Cutsem P. (1989). Influence of the degree of polymerization of oligogalacturonates and of esterification of pectin on their recognition by a monoclonal antibody. Plant Physiol. 91, 1419–1424. doi: 10.1104/pp.91.4.1419 16668976 PMC1080589

[B64] Lionetti V. Fabri E. De Caroli M. Hansen A. R. Willats W. G. T. Piro G. . (2017). Three pectin methylesterase inhibitors protect cell wall integrity for Arabidopsis immunity to Botrytis. Plant Physiol. 173, 1844–1863. doi: 10.1104/pp.16.01185 28082716 PMC5338656

[B65] Liu N. Zhang X. Sun Y. Wang P. Li X. Pei Y. . (2017). Molecular evidence for the involvement of a polygalacturonase-inhibiting protein, GhPGIP1, in enhanced resistance to Verticillium and Fusarium wilts in cotton. Sci. Rep. 7, 39840. doi: 10.1038/srep39840 28079053 PMC5228132

[B66] Malinovsky F. G. Fangel J. U. Willats W. G. T. (2014). The role of the cell wall in plant immunity. Front. Plant Sci. 5. doi: 10.3389/fpls.2014.00178 24834069 PMC4018530

[B67] Manabe Y. Nafisi M. Verhertbruggen Y. Orfila C. Gille S. Rautengarten C. . (2011). Loss-of-function mutation of REDUCED WALL ACETYLATION2 in Arabidopsis leads to reduced cell wall acetylation and increased resistance to *Botrytis cinerea*. Plant Physiol. 155, 1068–1078. doi: 10.1104/PP.110.168989 21212300 PMC3046569

[B68] Mansfield J. W. Richardson A. (1981). The ultrastructure of interactions between Botrytis species and broad bean leaves. Physiol. Plant Pathol. 19, 41–IN23. doi: 10.1016/S0048-4059(81)80006-9

[B69] Marcus S. E. Blake A. W. Benians T. A. S. Lee K. J. D. Poyser C. Donaldson L. . (2010). Restricted access of proteins to mannan polysaccharides in intact plant cell walls. Plant J. 64, 191–203. doi: 10.1111/j.1365-313X.2010.04319.x 20659281

[B70] Marcus S. E. Verhertbruggen Y. Hervé C. Ordaz-Ortiz J. J. Farkas V. Pedersen H. L. . (2008). Pectic homogalacturonan masks abundant sets of xyloglucan epitopes in plant cell walls. BMC Plant Biol. 8, 60. doi: 10.1186/1471-2229-8-60 18498625 PMC2409341

[B71] Mareri L. Romi M. Cai G. (2018). Arabinogalactan proteins: actors or spectators during abiotic and biotic stress in plants? Plant Biosyst. - Int. J. Dealing Aspects Plant Biosyst., 1–13. doi: 10.1080/11263504.2018.147352537339054

[B72] McCabe P. F. Valentine T. A. Forsberg L. S. Pennell R. I. (1997). Soluble signals from cells identified at the cell wall establish a developmental pathway in carrot. Plant Cell 9, 2225–2241. doi: 10.1105/tpc.9.12.2225 12237357 PMC157070

[B73] McCartney L. Marcus S. E. Knox J. P. (2005). Monoclonal antibodies to plant cell wall xylans and arabinoxylans. J. Histochem Cytochem 53, 543–546. doi: 10.1369/jhc.4B6578.2005 15805428

[B74] Meikle P. J. Bonig I. Hoogenraad N. J. Clarke A. E. Stone B. A. (1991). The location of (1→3)-β-glucans in the walls of pollen tubes of *Nicotiana alata* using a (1→3)-β-glucan-specific monoclonal antibody. Planta 185, 1–8. doi: 10.1007/BF00194507 24186272

[B75] Mendgen K. Hahn M. (2002). Plant infection and the establishment of fungal biotrophy. Trends Plant Sci. 7, 352–356. Available online at: http://ac.els-cdn.com/S1360138502022975/1-s2.0-S1360138502022975-main.pdf?_tid=211c40de-35d9-11e3-b46f-00000aab0f6c&acdnat=1381869423_0e2f9700eb5d611b3831b96edd514f2a. 10.1016/s1360-1385(02)02297-5 12167330

[B76] Merkouropoulos G. Shirsat A. H. (2003). The unusual Arabidopsis extensin gene atExt1 is expressed throughout plant development and is induced by a variety of biotic and abiotic stresses. Planta 217, 356–366. doi: 10.1007/s00425-003-1002-y 14520562

[B77] Moller I. Marcus S. E. Haeger A. Verhertbruggen Y. Verhoef R. Schols H. . (2008). High-throughput screening of monoclonal antibodies against plant cell wall glycans by hierarchical clustering of their carbohydrate microarray binding profiles. Glycoconjugate J. 25, 37–48. doi: 10.1007/s10719-007-9059-7 17629746 PMC2234451

[B78] Moller I. Sørensen I. Bernal A. J. Blaukopf C. Lee K. Øbro J. . (2007). High-throughput mapping of cell-wall polymers within and between plants using novel microarrays. Plant journal : For Cell Mol. Biol. 50, 1118–1128. doi: 10.1111/j.1365-313X.2007.03114.x 17565618

[B79] Murashige T. Skoog F. (1962). A revised medium for rapid growth and bio assays with tobacco tissue cultures. Physiol. Plant 15, 473–497. doi: 10.1111/j.1399-3054.1962.tb08052.x40046247

[B80] Nguema-Ona E. Moore J. P. Fagerström A. Fangel J. U. Willats W. G. T. Hugo A. . (2012). Profiling the main cell wall polysaccharides of tobacco leaves using high-throughput and fractionation techniques. Carbohydr Polym 88, 939–949. doi: 10.1016/j.carbpol.2012.01.044 24274496

[B81] Nguema-Ona E. Moore J. P. Fagerström A. D. Fangel J. U. Willats W. G. T. Hugo A. . (2013a). Overexpression of the grapevine PGIP1 in tobacco results in compositional changes in the leaf arabinoxyloglucan network in the absence of fungal infection. BMC Plant Biol. 13, 46. doi: 10.1186/1471-2229-13-46 23506352 PMC3621556

[B82] Nguema-Ona E. Vicré-Gibouin M. Cannesan M. A. Driouich A. (2013b). Arabinogalactan proteins in root-microbe interactions. Trends Plant Sci. 18, 1360–1385. doi: 10.1016/j.tplants.2013.03.006 23623239

[B83] Park Y. B. Cosgrove D. J. (2015). Xyloglucan and its interactions with other components of the growing cell wall. Plant Cell Physiol. 56, 180–194. doi: 10.1093/pcp/pcu204 25613914

[B84] Pedersen H. L. Fangel J. U. McCleary B. Ruzanski C. Rydahl M. G. Ralet M.-C. . (2012). Versatile high resolution oligosaccharide microarrays for plant glycobiology and cell wall research. J. Biol. Chem. 287, 39429–39438. doi: 10.1074/jbc.M112.396598 22988248 PMC3501085

[B85] Peng Y. van Wersch R. Zhang Y. (2018). Convergent and divergent signaling in PAMP-triggered immunity and effector-triggered immunity. Mol. Plant-Microbe Interact. 31, 403–409. doi: 10.1164/rccm.200909-1420OC 29135338

[B86] Plett J. M. Martin F. M. (2018). Know your enemy, embrace your friend: using omics to understand how plants respond differently to pathogenic and mutualistic microorganisms. Plant J. 93, 729–746. doi: 10.1111/tpj.13802 29265527

[B87] Popper Z. A. Fry S. C. (2005). Widespread occurrence of a covalent linkage between xyloglucan and acidic polysaccharides in suspension-cultured angiosperm cells. Ann. Bot. 96, 91–99. doi: 10.1093/aob/mci153 15837720 PMC4246812

[B88] Popper Z. A. Fry S. C. (2008). Xyloglucan-pectin linkages are formed intra-protoplasmically, contribute to wall-assembly, and remain stable in the cell wall. Planta 227, 781–794. doi: 10.1007/s00425-007-0656-2 17987313

[B89] Prins T. W. Tudzynski P. von Tiedemann A. Tudzynski B. Ten Have A. Hansen M. E. . (2000). “ Infection strategies of *Botrytis cinerea* and related necrotrophic pathogens,” in Fungal Pathology ( Springer Netherlands, Dordrecht), 33–64. doi: 10.1007/978-94-015-9546-9_2

[B90] Ralet M.-C. Crépeau M.-J. Vigouroux J. Tran J. Berger A. Sallé C. . (2016). Xylans provide the structural driving force for mucilage adhesion to the Arabidopsis seed coat. Plant Physiol. 171, 165–178. doi: 10.1104/pp.16.00211 26979331 PMC4854713

[B91] Ralet M.-C. Tranquet O. Poulain D. Moïse A. Guillon F. (2010). Monoclonal antibodies to rhamnogalacturonan I backbone. Planta 231, 1373–1383. doi: 10.1007/s00425-010-1116-y 20309579

[B92] Rashid A. (2016). Defense responses of plant cell wall non-catalytic proteins against pathogens. Physiol. Mol. Plant Pathol. 94, 38–46. doi: 10.1016/j.pmpp.2016.03.00942574925

[B93] Rijkenberg F. H. J. Leeuw G. T. N. D. Verhoeff K. (1980). Light and electron microscopy studies on the infection of tomato fruits by *Botrytis cinerea*. Can. J. Bot. 58, 1394–1404. doi: 10.1139/b80-17034819996

[B94] Rossi F. R. Krapp A. R. Bisaro F. Maiale S. J. Pieckenstain F. L. Carrillo N. (2017). Reactive oxygen species generated in chloroplasts contribute to tobacco leaf infection by the necrotrophic fungus *Botrytis cinerea*. Plant J. 92, 761–773. doi: 10.1111/tpj.13718 28906064

[B95] Ryder L. S. Talbot N. J. (2015). Regulation of appressorium development in pathogenic fungi. Curr. Opin. Plant Biol. 26, 8–13. doi: 10.1016/J.PBI.2015.05.013 26043436 PMC4781897

[B96] Saijo Y. Loo E. P. (2019). Plant immunity in signal integration between biotic and abiotic stress responses. New Phytol. 222(4), 1629–1637. doi: 10.1111/nph.15989 31209880

[B97] Saijo Y. Loo E. P. Yasuda S. (2018). Pattern recognition receptors and signaling in plant–microbe interactions. Plant J. 93, 592–613. doi: 10.1111/tpj.13808 29266555

[B98] Shah P. Powell A. L. T. T. Orlando R. Bergmann C. Gutierrez-Sanchez G. (2012). Proteomic analysis of ripening tomato fruit infected by *Botrytis cinerea*. J. Proteome Res. 11, 2178–2192. doi: 10.1021/pr200965c 22364583 PMC4874510

[B99] Sham A. Moustafa K. Al-Shamisi S. Alyan S. Iratni R. AbuQamar S. (2017). Microarray analysis of Arabidopsis WRKY33 mutants in response to the necrotrophic fungus *Botrytis cinerea*. PLoS One 12, e0172343. doi: 10.1371/journal.pone.0172343 28207847 PMC5313235

[B100] Shlezinger N. Minz A. Gur Y. Hatam I. Dagdas Y. F. Talbot N. J. . (2011). Anti-apoptotic machinery protects the necrotrophic fungus *Botrytis cinerea* from host-induced apoptotic-like cell death during plant infection. PLoS Pathog. 7, e1002185. doi: 10.1371/journal.ppat.1002185 21876671 PMC3158046

[B101] Showalter A. M. (1993). Structure and function of plant cell wall proteins. Plant Cell 5, 9–23. doi: 10.1105/tpc.5.1.9 8439747 PMC160246

[B102] Showalter A. M. (2001). Arabinogalactan-proteins: structure, expression and function. Cell Mol. Life Sci. 58, 1399–1417. doi: 10.1007/PL00000784 11693522 PMC11337269

[B103] Sicilia F. Fernandez-Recio J. Caprari C. De Lorenzo G. Tsernoglou D. Cervone F. . (2005). The polygalacturonase-inhibiting protein PGIP2 of *Phaseolus vulgaris* has evolved a mixed mode of inhibition of endopolygalacturonase PG1 of *Botrytis cinerea* 1. Plant Physiol. 139, 1380–1388. doi: 10.1104/pp.105.067546 16244152 PMC1283773

[B104] Smallwood M. Beven A. Donovan N. Neill S. J. Peart J. Roberts K. . (1994). Localization of cell wall proteins in relation to the developmental anatomy of the carrot root apex. Plant J. 5, 237–246. doi: 10.1046/j.1365-313X.1994.05020237.x37945311

[B105] Smallwood M. Martin H. Knox J. P. (1995). An epitope of rice threonine- and hydroxyproline-rich glycoprotein is common to cell wall and hydrophobic plasma-membrane glycoproteins. Planta 196, 510–522. doi: 10.1007/BF00203651 7544182

[B106] Smallwood M. Yates E. A. Willats W. G. T. Martin H. Knox J. P. (1996). Immunochemical comparison of membrane-associated and secreted arabinogalactan-proteins in rice and carrot. Planta 198, 452–459. doi: 10.1007/BF0062006330311153

[B107] Soltis N. E. Atwell S. Shi G. Fordyce R. Gwinner R. Gao D. . (2019). Interactions of tomato and *Botrytis cinerea* genetic diversity: Parsing the contributions of host differentiation, domestication, and pathogen variation. Plant Cell 31, 502–519. doi: 10.1105/tpc.18.00857 30647076 PMC6447006

[B108] Sowley E. N. K. K. Shaw M. W. Dewey F. M. Shaw M. W. (2009). Persistent, symptomless, systemic, and seed-borne infection of lettuce by *Botrytis cinerea*. Eur. J. Plant Pathol. 126, 61–71. doi: 10.1007/s10658-009-9524-130311153

[B109] Spadoni S. Zabotina O. Di Matteo A. Dalgaard Mikkelsen J. Cervone F. De Lorenzo G. . (2006). Polygalacturonase-inhibiting protein interacts with pectin through a binding site formed by four clustered residues of arginine and lysine 1. Plant Physiol. 141(2), 558–564. doi: 10.1104/pp.106.076950 16648220 PMC1475430

[B110] ten Have A. Breuil W. O. Wubben J. P. Visser J. van Kan J. A. L. (2001). *Botrytis cinerea* endopolygalacturonase genes are differentially expressed in various plant tissues. Fungal Genet. Biol. 33, 97–105. doi: 10.1006/FGBI.2001.1269 11456462

[B111] ten Have A. Tenberge K. Benen J. A. E. Tudzynski P. Visser J. van Kan J. A. L. (2002). “ The contribution of cell wall degrading enzymes to pathogenesis of fungal plant pathogens,” in Agricultural Applications ( Springer Berlin Heidelberg, Berlin, Heidelberg), 341–358. doi: 10.1007/978-3-662-03059-2_17

[B112] Tormo J. Lamed R. Chirino A. J. Morag E. Bayer E. A. Shoham Y. . (1996). Crystal structure of a bacterial family-III cellulose-binding domain: a general mechanism for attachment to cellulose. EMBO J. 15, 5739–5751. doi: 10.1002/j.1460-2075.1996.tb00960.x 8918451 PMC452321

[B113] Toruño T. Y. Stergiopoulos I. Coaker G. (2016). Plant-pathogen effectors: cellular probes interfering with plant defenses in spatial and temporal manners. Annu. Rev. Phytopathol. 54, 419–441. doi: 10.1146/annurev-phyto-080615-100204 27359369 PMC5283857

[B114] Trdá L. Boutrot F. Claverie J. Brulé D. Dorey S. Poinssot B. (2015). Perception of pathogenic or beneficial bacteria and their evasion of host immunity: pattern recognition receptors in the frontline. Front. Plant Sci. 6, 219. doi: 10.3389/fpls.2015.00219 25904927 PMC4389352

[B115] Tudzynski P. Kokkelink L. (2009). Botrytis cinerea: molecular aspects of a necrotrophic life style. Style DeKalb IL 5, 29–50. doi: 10.1007/978-3-540-87407-2_227457059

[B116] Valero-Jiménez C. A. Veloso J. Staats M. Van Kan J. A. L. (2019). Comparative genomics of plant pathogenic Botrytis species with distinct host specificity. BMC Genomics 20, 1–12. doi: 10.1186/s12864-019-5580-x 30866801 PMC6417074

[B117] Van der Does D. Boutrot F. Engelsdorf T. Rhodes J. McKenna J. F. Vernhettes S. . (2017). The Arabidopsis leucine-rich repeat receptor kinase MIK2/LRR-KISS connects cell wall integrity sensing, root growth and response to abiotic and biotic stresses. PLoS Genet. 13, e1006832. doi: 10.1371/journal.pgen.1006832 28604776 PMC5484538

[B118] van Kan J. A. L. (2005). Infection strategies of *Botrytis cinerea*. Acta Hortic. 669, 77–90. doi: 10.1007/978-94-015-9546-930311153

[B119] van Kan J. A. L. (2006). Licensed to kill: The lifestyle of a necrotrophic plant pathogen. Trends Plant Sci. 11, 247–253. doi: 10.1016/j.tplants.2006.03.005 16616579

[B120] van Kan J. A. L. Shaw M. W. Grant-downton R. T. (2014). Botrytis species: relentless necrotrophic thugs or endophytes gone rogue? 15, 957–961. doi: 10.1111/mpp.12148 PMC6638755 24754470

[B121] Veloso J. van Kan J. A. L. (2018). Many shades of grey in Botrytis–host plant interactions. Trends Plant Sci. 23, 613–622. doi: 10.1016/j.tplants.2018.03.016 29724660

[B122] Verhertbruggen Y. Marcus S. E. Haeger A. Ordaz-Ortiz J. J. Knox J. P. (2009a). An extended set of monoclonal antibodies to pectic homogalacturonan. Carbohydr Res. 344, 1858–1862. doi: 10.1016/j.carres.2008.11.010 19144326

[B123] Verhertbruggen Y. Marcus S. E. Haeger A. Verhoef R. Schols H. A. McCleary B. V. . (2009b). Developmental complexity of arabinan polysaccharides and their processing in plant cell walls. Plant J. 59, 413–425. doi: 10.1111/j.1365-313X.2009.03876.x 19392693

[B124] Vorwerk S. Somerville S. Somerville C. (2004). The role of plant cell wall polysaccharide composition in disease resistance. Trends Plant Sci. 9, 203–209. doi: 10.1016/j.tplants.2004.02.005 15063871

[B125] Wei G. Shirsat A. H. (2006). Extensin over-expression in Arabidopsis limits pathogen invasiveness. Mol. Plant Pathol. 7, 579–592. doi: 10.1111/j.1364-3703.2006.00363.x 20507471

[B126] Willats W. G. T. Marcus S. E. Knox J. P. (1998). Generation of a monoclonal antibody specific to (1→5)-α-l-arabinan. Carbohydr Res. 308, 149–152. doi: 10.1016/S0008-6215(98)00070-6 9675359

[B127] Willats W. G. McCartney L. Mackie W. Knox J. P. (2004). Functional and molecular characterization of a monoclonal antibody to xylogalacturonan. Planta 218, 673–681. doi: 10.1007/s00425-003-1157-z30311153

[B128] Williamson B. Tudzynski B. Tudzynski P. van Kan J. A. L. (2007). *Botrytis cinerea*: The cause of grey mould disease. Mol. Plant 8, 561–580. doi: 10.1111/J.1364-3703.2007.00417.X 20507522

[B129] Wolf S. (2017). Plant cell wall signalling and receptor-like kinases. Biochem. J. 474, 471–492. doi: 10.1042/BCJ20160238 28159895

[B130] Wu Y. Fan W. Li X. Chen H. Takáč T. Šamajová O. . (2017). Expression and distribution of extensins and AGPs in susceptible and resistant banana cultivars in response to wounding and Fusarium oxysporum. Sci. Rep. 7, 42400. doi: 10.1038/srep42400 28218299 PMC5316987

[B131] Wubben J. P. ten Have A. van Kan J. A. L. Visser J. (2000). Regulation of endopolygalacturonase gene expression in *Botrytis cinerea* by galacturonic acid, ambient pH and carbon catabolite repression. Curr. Genet. 37, 152–157. doi: 10.1007/s002940050022 10743572

[B132] Yates E. A. Valdor J.-F. Haslam S. M. Morris H. R. Dell A. Mackie W. . (1996). Characterization of carbohydrate structural features recognized by anti-arabinogalactan-protein monoclonal antibodies. Glycobiology 6, 131–139. doi: 10.1093/glycob/6.2.131 8727785

[B133] York W. S. Darvill A. G. McNeil M. Stevenson T. T. Albersheim P. (1986). Isolation and characterization of plant cell walls and cell wall components 3–40. doi: 10.1016/0076-6879(86)18062-1

[B134] Zhang W. Corwin J. A. Copeland D. Feusier J. Eshbaugh R. Chen F. . (2017). Plastic transcriptomes stabilize immunity to pathogen diversity: The jasmonic acid and salicylic acid networks within the Arabidopsis/Botrytis pathosystem. Plant Cell 29, 2727–2752. doi: 10.1105/tpc.17.00348 29042403 PMC5728128

[B135] Zhu W. Ronen M. Gur Y. Minz-Dub A. Masrati G. Ben-Tal N. . (2017). BcXYG1, a secreted xyloglucanase from Botrytis cinerea, triggers both cell death and plant immune responses. Plant Physiol. 175, 438–456. doi: 10.1104/pp.17.00375 28710128 PMC5580746

[B136] Zykwinska A. Thibault J.-F. Ralet M.-C. (2007). Organization of pectic arabinan and galactan side chains in association with cellulose microfibrils in primary cell walls and related models envisaged. J. Exp. Bot. 58, 1795–1802. doi: 10.1093/jxb/erm037 17383990

