# Supplementary material for: Overexpression of the grapevine PGIP1 in tobacco is associated with leaf cell wall pectin modification and glycoprotein changes following Botrytis cinerea infection
Source: Front Plant Sci. 2026 Oct 21;17:1935736. doi: 10.3389/fpls.2026.1935736 (PMC13731287; doi:10.3389/fpls.2026.1935736)
Supplement: Supplementary file 1 [file DataSheet1.docx]

Supplementary Material

# 1 Supplementary Figures

## 1.1 Supplementary Figures

**Supplementary Figure 1:** CDTA fraction of a comprehensive microarray polymer profiling (CoMPP) analysis of the wild-type SR1, PGIP37 and PGIP45 tobacco leave AIR of adjacent tissue to the infection. Results are presented as heatmaps showing the relative abundance of cell wall associated epitopes (see material and methods for more information). A detailed list of the antibodies used is available in Table 1.

**Supplementary Figure 2:** NaOH fraction of a comprehensive microarray polymer profiling (CoMPP) analysis of the wild-type SR1, PGIP37 and PGIP45 tobacco leave AIR of adjacent tissue to the infection. Results are presented as heatmaps showing the relative abundance of cell wall associated epitopes (see material and methods for more information). A detailed list of the antibodies used is available in Table 1.

**Supplementary Figure 3:** CDTA fraction of a comprehensive microarray polymer profiling (CoMPP) analysis of the wild-type SR1, PGIP37 and PGIP45 tobacco leave AIR of lesions. Results are presented as heatmaps showing the relative abundance of cell wall associated epitopes (see material and methods for more information). A detailed list of the antibodies used is available in Table 1.

**Supplementary Figure 4:** NaOH fraction of a comprehensive microarray polymer profiling (CoMPP) analysis of the wild-type SR1, PGIP37 and PGIP45 tobacco leave AIR of lesions. Results are presented as heatmaps showing the relative abundance of cell wall associated epitopes (see material and methods for more information). A detailed list of the antibodies used is available in Table 1.

**Supplementary Figure 5:** Principal component analysis (PCA) of CoMPP results of AIR from SR1, PGIP37 and PGIP45 non-infected tobacco leave tissue (adjacent and distal to the infection). A, B: PCA showing sample separation for the CDTA (A) and NaOH (B) extractions. Samples are coloured according to the time after Botrytis spore inoculation C, D: Loading plots of the respective PCA showing the epitopes that drive sample separation. Interesting groupings of antibodies observed on the loading plots are circled in blue. A detailed list of the antibodies used is available in Table 1.

**Supplementary Figure 6:** Principal component analysis (PCA) of CoMPP results of AIR from SR1, PGIP37 and PGIP45 infected tissue (lesion). A, B: PCA showing sample separation for the CDTA (A) and NaOH (B) extractions. Samples are coloured according to the time after Botrytis spore inoculation (C, D): Loading plots of the respective PCA showing the epitopes that drive sample separation. Interesting groupings of antibodies observed on the loading plots are circled in blue. A detailed list of the antibodies used is available in Table 1.
